# Immune checkpoint therapy for solid tumours: clinical dilemmas and future trends

**DOI:** 10.1038/s41392-023-01522-4

**Published:** 2023-08-28

**Authors:** Qian Sun, Zhenya Hong, Cong Zhang, Liangliang Wang, Zhiqiang Han, Ding Ma

**Affiliations:** 1 Department of Obstetrics and Gynecology, Tongji Hospital, Tongji Medical College, Huazhong University of Science and Technology, Wuhan, Hubei 430030 China; 2 Cancer Biology Research Center (Key Laboratory of the Ministry of Education), Tongji Hospital, Tongji Medical College, Huazhong University of Science and Technology, Wuhan, Hubei 430030 China; 3 Department of Hematology, Tongji Hospital, Tongji Medical College, Huazhong University of Science and Technology, Wuhan, Hubei 430030 China

**Keywords:** Tumour immunology, Drug development

## Abstract

Immune-checkpoint inhibitors (ICBs), in addition to targeting CTLA-4, PD-1, and PD-L1, novel targeting LAG-3 drugs have also been approved in clinical application. With the widespread use of the drug, we must deeply analyze the dilemma of the agents and seek a breakthrough in the treatment prospect. Over the past decades, these agents have demonstrated dramatic efficacy, especially in patients with melanoma and non-small cell lung cancer (NSCLC). Nonetheless, in the field of a broad concept of solid tumours, non-specific indications, inseparable immune response and side effects, unconfirmed progressive disease, and complex regulatory networks of immune resistance are four barriers that limit its widespread application. Fortunately, the successful clinical trials of novel ICB agents and combination therapies, the advent of the era of oncolytic virus gene editing, and the breakthrough of the technical barriers of mRNA vaccines and nano-delivery systems have made remarkable breakthroughs currently. In this review, we enumerate the mechanisms of each immune checkpoint targets, associations between ICB with tumour mutation burden, key immune regulatory or resistance signalling pathways, the specific clinical evidence of the efficacy of classical targets and new targets among different tumour types and put forward dialectical thoughts on drug safety. Finally, we discuss the importance of accurate triage of ICB based on recent advances in predictive biomarkers and diagnostic testing techniques.

## Introduction

Immune checkpoint blockade (ICB) drugs have been initially approved as early as 2011 for the treatment of unresectable advanced melanoma after classical therapy.^1^ In the past decade, ICBs have made a great breakthrough in the field of advanced cancer treatment, gradually moving from second or third-line drugs to first-line drugs. As novel co-inhibitory and co-stimulatory receptors are gradually discovered and explored, it is necessary to have a comprehensive understanding of the members of these new ICB targets, and identify the most potential targets that can be transformed into clinical practice and bring clinical benefits. The key signalling pathways and regulatory mechanisms are the core scientific issues for deeply understanding the principles of ICB treatment and seeking future development directions. Through extensive literature review and analysis, we hope to find the specific signalling pathways related to ICB reactivity, side effects and drug resistance from the gene mutations of tumour cells and the intricate regulatory network of immune cells, to clarify the future trends of ICB development.

As of September 2022, nine drugs targeting four immune checkpoints, cytotoxic T-lymphocyte associated protein-4 (CTLA-4), programmed cell death-1 (PD-1), programmed death ligand-1(PD-L1) and lymphocyte activation gene-3 (LAG-3) have already been approved by the US Food and Drug Administration (FDA). In view of the widespread use of ICB in solid tumours, we are facing many dilemmas in clinical use that need to be solved. Due to the imperfect indications and efficacy evaluation system, it is difficult to precisely determine the starting and ending time of ICB immunotherapy intervention. Firstly, the reasons for the substantial variation in treatment response among different tumours are not fully understood which exposes patients who do not respond to ICB treatment to the risk of adverse effects. Secondly, it is difficult to distinguish unconfirmed progressive disease (hyper progression or pseudoprogression) in the early stage, which can lead to premature or late discontinuation of treatment. More importantly, in the face of a large number of drug-resistant people and severe adverse effects, we need to propose feasible plans for improving the efficacy of ICB treatment and standardize the application of ICB.

In this review, we cover all the immune checkpoint molecules and highlight their related signalling pathways/regulatory mechanisms at the beginning. Then, the current progress of ICB treatment is discussed point by point, including tumour gene mutation, drug resistance mechanism, combination therapy, and the latest preclinical and clinical trial advances. In addtion, we summarize the clinical dilemmas and solutions of ICB from the following four aspects: broad spectrum indications for ICB, inseparable immune response and immune-related adverse effects, the elusive unconfirmed progressive disease, research progress on predictive biomarkers and imaging examination.

## An overview of immune checkpoints molecules and related signalling pathways

**Fig. 1:**
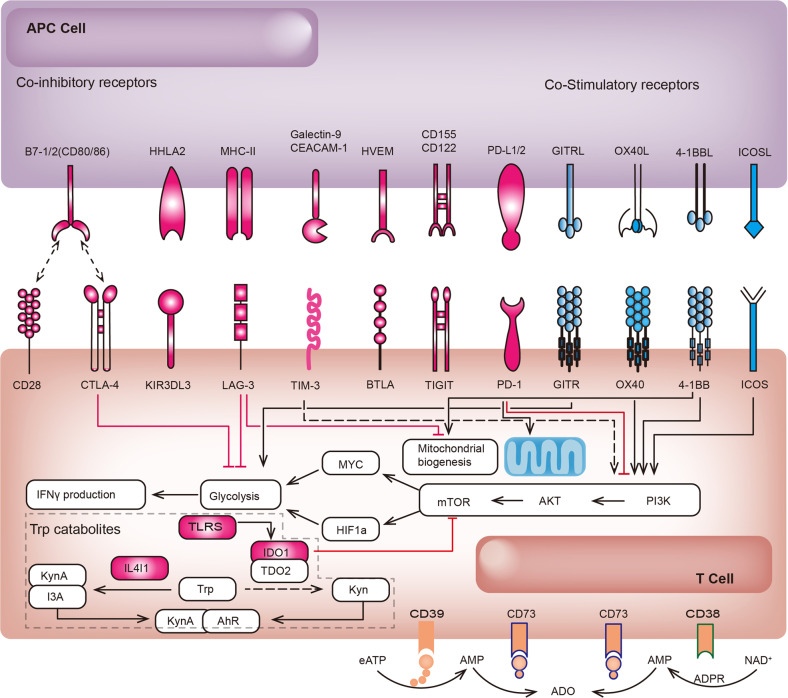
Interaction of novel immune checkpoint receptors and their respective ligands. Cytotoxic T-lymphocyte-associated antigen-4 (CTLA-4) competitively binds to CD80/86 and limits initial T cell activation; programmed cell death protein-1 (PD-1) binds to PD-L1 and inhibits effector T activation and expansion; lymphocyte activation gene-3 (LAG-3) competitively binds to major histocompatibility complex II (MHC II) and inhibits effector T cell activation; T cell immunoglobulin and mucin domain 3 (TIM-3) binds to carcinoembryonic antigen cell adhesion molecule 1 (CEACAM-1) or Galectin-9 and triggers CD8^+^ T cell exhaustion; B and T Lymphocyte Attenuator (BTLA) binds to Herpesvirus entry mediator (HVEM) and suppresses TCR signalling; T cell Immunoglobulin and ITIM domain (TIGIT) binds to CD122 or CD155 and downregulates cell functions of T cells and NK cells;, Co-stimulatory receptors include inducible costimulatory molecule (ICOS), glucocorticoid-induced TNF receptor family-related protein (GITR), TNF receptor superfamily member 4 (OX40), and TNF receptor superfamily member 9 (4-1BB). Tryptophan (Trp) catabolism molecules (IL4I1 and IDO-1) and adenosine signalling molecules (CD39 and CD73) are also involved

With the application of ICBs, scientists have gradually realized that the immune activation generated by targeting CTLA-4 or PD-1 is not sufficient to control tumour progression. Consequently, research to explore novel targets based on diversified intervention pathways is progressing rapidly. Based on the conclusions of the current preclinical and clinical studies, we can preliminarily see that the most promising targetable co-inhibitory receptors include but are not limited to LAG-3, T cell immunoglobulin and ITIM domain (TIGIT), T cell immunoglobulin and mucin-domain containing-3 (TIM-3), indoleamine 2,3-dioxygenase (IDO), CD39, NKG2A, and signal regulatory protein alpha (SIRPα). Co-stimulatory receptors include inducible T cell costimulator (ICOS), glucocorticoid-induced TNF receptor family-related protein (GITR), TNF receptor superfamily member 4 (OX40), TNF receptor superfamily member 9 (4-1BB), and Toll-like receptors (TLRs).^2^ We elaborate on the immunomodulatory and antitumour immune mechanisms/signalling pathways of the first-generation ICB targeting CTLA-4, the second-generation ICB targeting PD-1/PD-L1, novel co-inhibitory receptors and co-stimulatory receptors successively. The network of mutual regulatory mechanisms of these immune checkpoints is more clearly described schematically (Fig. 1).

### Molecular mechanisms of the first-generation of ICB targeting CTLA-4

CTLA-4, encodes inhibitory proteins participating in T cell stimulation. There is a competitive binding relationship between CTLA-4 and CD28, but CTLA-4 is more advantageous because of its 100-fold affinity to CD80/CD86 ligands,.^3^ CD28 is stably expressed on almost entire CD4^+^ T cells and nearly half of CD8^+^ T cells, while the expression of CTLA-4 is very finite. The balance of CTLA-4 trafficking to the cell membrane and rapid internalization is crucial for T cell activation.^4^ T-cell receptor-interacting molecule (TRIM) binds to CTLA-4 and transports to the cell membrane, which will activate phosphatidylinositol 3 kinases (PI3K) and protein kinase B (PKB)/AKT signalling pathways and lead to T cell dysfunction.^5^ On the contrary, CTLA-4 is highly expressed on regulatory T cells (Tregs) and downregulates the CD80/CD86 molecules on the surface of antigen-presenting cells (APCs) via trogocytosis. Furthermore, an increasing of free PD-L1 on dendritic cells (DCs) occurs at the same time. This is theoretical evidence that combined CTLA-4 and PD-Ll target blockade can reduce Treg-mediated immune suppression.^6^ CTLA-4 may also inhibit T cell responses by generating inhibitory signals, inhibiting CD8^+^ T cells,^7^ stimulating the IDO pathway,^8^ and affecting T helper cell differentiation,^9^ affecting protein trafficking.^10–12^ Due to the multiple roles of CTLA-4 in initiating and mounting T cell responses, there are many internal regulatory mechanisms in the internal environment.^13^ For instance, the nuclear factor of activated T cells (NFAT) and forkhead box P3 (FOXP3) interact with each other and promotes CTLA-4 transcription at RNA transcriptional level.^14,15^ Inhibition of NFAT/FOXP3 interaction may improve T cell response to TCR stimulation pathway.^16^ On the contrary, disruption of feedback loop of CTLA-4 and CD28 may enhance Treg cell expansion.^17^ The non-unidirectional effect on the regulation of T cell function may make its clinical application difficult to control.

Heterozygous germline mutations in CTLA-4 will cause complex immune dysregulation syndrome which suggests an indispensable role of this gene in maintaining homoeostasis.^18^ Fortunately, some advanced progress has gradually confirmed the feasibility of conditional blockade. For instance, anti-CTLA-4 IgG2a antibodies may reduce intratumoral Treg cells.^19^ However, the loss of Treg surface expressed CTLA-4 may promote ICB-related adverse events (irAEs). PH-sensitive anti-CTLA-4 antibodies participate in CTLA-4 recycling which enables CTLA-4 and LRBA binding complex and re-expression of CTLA-4 on Treg cell surface.^20^ In addition, conditionally active anti-CTLA-4 antibodies engineered with protein-associated chemical switches (PaCS) can help to improve tumour cell-specific blockade and reduce binding to normal cells.^21^ Moreover, MED15752, a monovalent bispecific DuetMab antibody, has been identified to bind with CTLA-4 on PD-1 activated T cells specifically.^22^ Several other combination approaches can enhance the positive immunotherapy effect of CTLA-4. A combination of SRC family kinases inhibition with CTLA-4 blockade may increase the immunotherapy efficacy in head and neck squamous cell carcinoma (HNSCC).^23^ Interleukin 2 (IL-2) combined with anti-CTLA-4 and anti-PD-1 can induce tumour specific CD8^+^ T cell expansion and overcome immunological resistance.^24^ In summary, CTLA-4, as the target of the earliest generation of immune checkpoint inhibitors, still has a large space for exploration in the tumour immune regulatory network.

### Molecular mechanisms of the second-generation of ICB targeting PD-1 or PD-L1

PD-1 (encoded by *CD279*) has two ligands, PD-L1 (encoded by *CD274*) and PD-L2 (encoded by *CD273*).^25^ PD-L1 is expressed on the surface of most hematopoietic or non-hematopoietic cells, while PD-L2 is expressed on macrophages, DCs and non-hematopoietic lung cells. Upon PD-1 binding with its ligands, Src homology region 2 domain-containing phosphatases (SHP-2) recruit to the ITIM and ITSM tyrosine motifs, resulting in dephosphorylation of TCR signalling molecules and T cell activated inhibition.^26,27^ PD-1 is inducibly expressed in most types of immune cells. For example, PD-1 expression on T cells can be induced via TCR signalling and type I IFN signalling.^28^ PD-1 is upregulated during CD8^+^ T cell activation after chronic virus infection through loss of DNA methylation of Pdcd1.^29^ H3K4me3 binds to the Pdcd1 gene promoter in activated CD4^+^ and CD8^+^ T cells.^30^ The NFAT2 and STAT regulatory regions bind to the promoter region of the Pdcd1 gene and promote PD-1 transcription at RNA transcriptional level. Meanwhile, proinflammatory cytokines (IL-6 or IL-12) can augment the expression of PD-1 through activating STAT3/STAT4 pathway.^31^ Activated CD4^+^ T cells undergoing oxidative phosphorylation exhibit higher PD-1 expression.^32^ Cytokines and TLR/NF-κB signalling also induce PD-1 expression in macrophages.^33^

PD-L1 expression can be induced by both types I and II IFNs, IL-10, and TNF-α on microvascular endothelial cells.^34^ PD-L1 expressing macrophages and myeloid derived suppressor cells (MDSCs) can be induced with the coculture of bladder tumour cells through COX2/mPGES1/PGE2 pathway.^35^ The studies on the regulation of PD-L1 expression among different tumour cells seem to get more attention. The regulation mechanism is complicated, including various processes in gene transcription and post-transcriptional modifications (PTMs).^36^ At the level of DNA regulation, genomic alteration and epigenetic regulation of histone acetylation and methylation are involved. Histone deacetylase 6 (HDAC6) has been identified in the activation of STAT3 and upregulation of PD-L1 in melanoma.^37,38^ H3K4me3 can be catalyzed a promote PD-L1 transcription in pancreatic cancer and melanoma.^39–41^ At the level of RNA regulation, inflammatory signalling, aberrant oncogenic signalling and indirect regulation by miRNA are summarized. In brief, epidermal growth factor receptor (EGFR) signalling, mitogen-activated protein kinase (MAPK) signalling, PTEN/PI3K/AKT signalling pathway, JAK-STAT signalling pathway, NF-κB signalling pathway, HIF-1α signalling pathways are key aberrant oncogenic signallings which will be discussed in the section on mechanisms of drug resistance in detail.^13,36^

As the most concerned ICB, anti-PD-1/anti-PD-L1 drugs are designed to bind with cell-surface immune checkpoints and block immunosuppressive pathways. When anti-PD-1 binds with PD-1 on the T cell membrane surface, or anti-PD-L1 binds with PD-L1 on the tumour cell and antigen-presenting cell (APC) membrane surface, naïve T cell will be activated, expanded, and released perforin and granzyme which causing enhanced tumour killing.^42^ In parallel, conditional deletion or blockade of PD-1 in Treg cells can enhance antitumour immunity through weakening Treg cell proliferation and infiltration in the tumour microenvironment.^43^ The evidence of ICB efficacy targeting PD-1/PD-L1 in clinical trials has become discussed among multiple cancers. We will discuss the latest progress and clinical dilemma of ICB application in the section on clinical trials.

### Novel co-inhibitory receptors

LAG-3, also known as CD223, is a single-pass transmembrane glycoprotein and is structurally homologous to CD4.^44^ LAG-3, with a higher affinity than CD4, can negatively regulate conventional CD4^+^ T cells through competitively binding to major histocompatibility complex class II (MHC II) on APC cell surface.^45–47^ On the other hand, LAG-3 expressing NK cells and CD8^+^ T cells can be affected through binding with LSECtin on liver cells and many tumour cells.^48^ Despite three decades of research, we still have many unanswered questions about the structure and function of LAG-3. For instance, the high-resolution structure of LAG-3 remains undeciphered, the signal transduction mechanism of LAG-3 and its ligands is still being explored, and the imperfect preclinical models and differences in gene expression values in different clinical tissues are still puzzling us.^49^ There are multiple LAG-3 targeted therapy subtypes: anti-LAG-3 monoclonal antibodies, LAG-3-immunoglobulin fusion proteins, and LAG-3 bispecifics.^50^ The first FDA-approved drug of LAG-3 inhibitor is named OPDUALAG, which is a combination of anti-LAG-3 antibody (relatlimab-rmbw) and anti-PD-1 antibody (nivolumab). The drug efficacy and safety have been investigated in phase 2–3 clinical trials with 714 untreated metastatic or unresectable melanoma patients. The clinical benefit of the median progression-free survival (PFS) has been increased from 4.6 months (nivolumab) to 10.1 months (relatlimab and nivolumab). Meanwhile, the adverse event occurrence (Grade≥3) has increased from 9.7% (nivolumab) to 18.9% (relatlimab and nivolumab) (NCT03470922).^51^ Another current phase 3 trial evaluating the efficacy of LAG-3 blocking antibody (MK-4280 and REGN3767) and LAG-3 bispecific (MGD013) in HER2^+^ gastric and gastroesophageal junction cancer patients (NCT04082364), PD-1^+^ colorectal cancer patients (NCT05064059) and melanoma (NCT05352672) are ongoing.

TIGIT, acts as a ligand for the poliovirus receptor (PVR) family members (CD122, CD155) or a competitive inhibitor for CD226. TIGIT/CD226 receptor pair, whose relationship is analogous to the CTLA-4/CD28 receptor pair, fulfils the role of co-inhibitory or co-stimulatory function.^52^ TIGIT can inhibit NK cell-mediated tumour killing and induce immunosuppressive DCs.^53^ The dual blockade of the TIGIT and PD-1 axis stimulates the effective T cells and NK cells in ovarian cancer patients.^54^ The most promising anti-TIGIT mAbs include tiragolumab (GO30103), zimberelimab (AB122), and tislelizumab (BGB-A317). In the CITYSCAPE trial, median PFS increased from 3.6 months (atezolizumab) to 5.4 months (tiragolumab plus atezolizumab) in 135 NSCLC patients. Meanwhile, serious adverse event occurrence has increased from 18.0% (atezolizumab) to 21.0% (tiragolumab plus atezolizumab).^55^ There are a series of phase 3 clinical trials of anti-TIGIT plus anti-PD-L1 or anti-PD-1 in patients with lung cancer, ESCC, and upper gastrointestinal tract adenocarcinoma.^56^

TIM-3, also known as hepatitis A virus cellular receptor 2 (HAVCR2), is an inhibitory molecule involved in tolerance and overexpressed in exhausted T cells. CD8^+^ T cell apoptosis or exhaustion will be triggered when TIM-3 combines with carcinoembryonic antigen cell adhesion molecule 1 (CEACAM-1) or galectin 9 (Gal-9).^57,58^ PD-1 interacts with Gal-9 can inhibit the progressed T cell death and promote CD8^+^ T cell expansion.^59^ In addition to regulating T cells, blockade of Gal-9/TIM-3 signalling may also inhibit macrophage M2 polarization and glioma growth.^60^ The antitumour response of sabatolimab (MBG453), a mAb that inhibits TIM-3 checkpoint, has been observed in phase 1/2 clinical trials.^61^ A randomized, double-blind, placebo-controlled phase 3 study of MBG453 is currently active in 530 patients with myelodysplastic or chronic myelomonocytic leukaemia-2 (NCT04266301).

IDO is a heme-containing enzyme participating in tryptophan catabolism. IDO-expressing DCs, monocytes, and macrophages can modulate T cell regulation and acquired tolerance.^62^ IDO-expressing DC can activate Treg cells by regulating mTORC2 and Akt signalling pathways.^63^ Tumour cells express IDO during tumourigenesis and induce tumour tolerance in anti-tumour immunity via activating STING/IFNaβ signalling.^64,65^ Four types of IDO1 inhibitors are Type I binding to oxygen-bound holo-IDO1, Type II binding to free ferrous holo-IDO1 (e.g.epacadostat), Type III binding to free ferric holo-IDO1 (e.g.navoximod) and Type IV binding to apo-IDO1 (e.g. lnrodosta).^66^ The combination of epacadostat plus pembrolizumab has got encouraging results in the phase 1/2 trial in patients with advanced solid tumours, while the large phase 3 trial failed with no improvement in PFS and OS compared to pembrolizumab monotherapy.^67,68^ Due to the exact role of IDO in antitumour immunity, multiple clinical trials are still undergoing highly anticipated.^69^

Interleukin-4-induced-1 (IL4I1) expression is higher than IDO1 in most cancer types. The expression of AHR target genes was not affected by hypoxia in IL4I1-overexpressing cells, indicating that IL4I1 can also activate aryl hydrocarbon receptor (AHR) in hypoxic (TME).^70,71^ IL4I1 can improve PD-L1 expression in lung adenocarcinoma through JAK/STAT pathway.^72^ In addition, IL4I1 overexpression will suppress the function of CTL and enhance T cell exhaustion in colorectal cancer.^73^ The preclinical evidence supports IL4I1 as a novel target for novel immune checkpoint inhibition.

CD39, encoded by ectonucleoside triphosphate diphosphohydrolase 1 (ENTPD1), is a membrane protein participating in hydrolyzing extracellular ATP to AMP. Meanwhile, CD39 also increases extracellular adenosine production which is hydrolyzed via CD73. As key rate-limiting enzymes of the adenosinergic pathway, expressions of CD39 and CD73 will limit immune responses to inflammatory signals in the tumour microenvironment (TME), and affect the proliferation of tumours indirectly.^74–76^ However, the expression status of the two enzymes in TME is not the same. CD39 and CD73 are both expressed on vascular endothelial cells and fibroblasts. Specifically, CD39 are more frequently expressed in immune cells, while CD73 is expressed in tumour cells and myeloid cells. Multiple progresses including accumulation of eATP and decrease of adenosine (ADO) will occur within the TME after CD39 inhibition. As a result, increased eATP activates pro-inflammatory cytokines release and enhances antigen presentation and mutaration.^77–79^ Most clinical trials of CD39 or CD73 inhibitors are under recruiting status currently.

NKG2A, encoded by killer cell lectin-like receptor C1 (KLRC1), belongs to the KLRC (also known as NKG2) family and is expressed in natural kill (NK) cells. The complex of NKG2A binding with CD94 participates in the recognition of the MHC I HLA-E molecules.^80^ Blockade of the immune checkpoint pair will enhance NK-mediated tumour cell killing.^81^ In addition, enriched interaction of NKG2A and CD94 will also reduce tumour infiltration of CD8^+^ T cells in the TME and can be reversed by blocking agents.^82–84^ Monalizumab is a humanized IgG4 NKG2A-blocking antibody which has been administered in phase 2 clinical trials.^85^ Monalizumab monotherapy has limited efficacy in a cohort of twenty-six HNSCC patients.^86^ Whereas, the result of COAST (NCT03822351) shows prolonged PFS with a combination of monalizumab plus durvalumab than durvalumab in unresectable stage III NSCLC patients.^87^

SIRPα is a member of the SIRP family participating in the negative regulation of tyrosine kinase. SIRPα-expressing myeloid cells bind to CD47-expressing tumour cells and mediate negative regulation of cytotoxicity of macrophage, neutrophil and microglia cells towards cancer.^88^ Blockade of the SIRPα-CD47 axis will increase phagocytosis of macrophages.^89^ Hu5F9-G4 (magrolimab), a humanized antibody against CD47, has been used in preclinical research among HER2-positive breast cancer patients combined with trastuzumab.^90^

### Novel co-stimulatory receptors

ICOS, also known as AILIM, CD278, or CVID1, promotes TCR co-stimulation and Treg cell stimulation.^91^ A randomized, parallel-group, phase 2/3 study with feladilimab (GSK3359609), a mAb that inhibits ICOS checkpoint, is completed in PD-L1 positive head and neck squamous cell carcinoma (NCT04128696). The clinical benefit with the significant difference of this combination treatment has not been observed. The frequency of serious adverse events for participants receiving feladilimab and pembrolizumab is 28.9%. The phase 1 clinical trial of TRX518 (anti-GITR mAb) has been completed in ten stages III or IV melanoma or other solid tumour patients without results (NCT01239134). Another phase 2 study of anti-GITR agonist INCAGN1876 combined with PD-1 inhibitor INCMGA00012 in recurrent glioblastoma is ongoing (NCT04225039). The phase 2 clinical trial of BMS-986178 (OX40 agonist) alone or combined with nivolumab/ ipilimumab in advanced solid tumour patients has been completed. The safety of the OX40 agonist has been confirmed but additional clinical efficacy has not been observed (NCT02737475).^92^ Another promising OX40 agonist (INCAGN01949) has been conducted in 87 advanced or metastatic solid tumour patients (NCT02923349). Similarly, a limited response has been observed in phase 2 clinical trial.^93^ Although the follow-up evaluation of overall survival (OS) and long-term benefit in these trials are incomplete, the conclusions give us great encouragement for dual checkpoint inhibitor exploration.

TLRs are expressed widely in the TME and have generated our interest to regulate TLRs as immune checkpoint molecules. TLRs can promote tumour progression through activating NF-κB signalling, inducing immunosuppressive cytokines and IDO production.^94,95^ DAMPs and PAMPs in the TME can activate the TLR signalling pathway and lead to tumour metastasis and chemoresistance. Moreover, TLR signalling within CAFs and MDSCs can be induced and lead to pro-tumoural effects.^96^ In contrast, TLRs also elicit antitumoural responses within immune cells. Generally, TLR agonists are very promising for tumour immune therapy.^97^ Preclinical and phase 1 clinical trial results of a TLR7 agonist have been identified with induction of type I IFN responses in HER2^+^ solid tumour patients (NCT03696771).^98^ In addition, TLR7/8 agonist has been attempted to use combined with ICB and enhances immunity response in TME.^99–101^

## ICB and tumour gene mutations

### Tumour gene mutations associated with ICB response

**Fig. 2:**
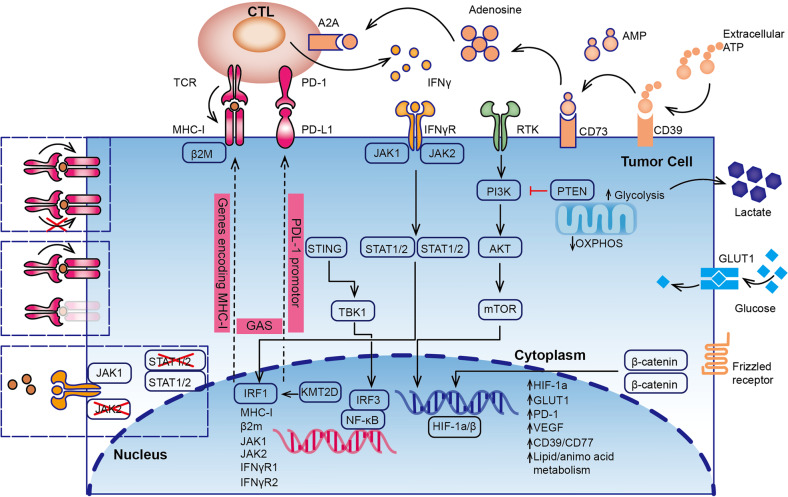
Important genes signalling pathways and metabolic alterations associated with ICB responsiveness or resistance in tumour cells. Anti-tumour immunity is mainly through the killing effect of CTL on tumour cells. Tumour gene mutations involved in the PI3K-Akt-mTOR axis, hypoxia-inducible factor-1α (HIF-1α) pathway, Janus kinase (JAK)/signal transducer and activator of transcription (STAT) pathway, and NF-κB pathways will affect PD-1 blockade responsiveness and resistance. Metabolic alterations of glycolysis, aggressive depletion of amino acids, and immune-suppressive productions are essential factors influencing CTL anergy and ICB resistance

High tumour mutation burden (TMB-H) has been utilized as a predictive biomarker for ICB response, due to the potential of neoantigens generation during tumour gene mutations. More gene mutations are superior than TMB-H in predicting response to pembrolizumab with NSCLC.^102^ Recurrent somatic mutations of BCLAF1, KRAS, BRAF, and P53, as well as MAPK signalling and p53-associated pathways, are predictors of ICB response.^103^ Metabolic dysregulation in tumour cells also has a great impact on the efficacy of immunotherapy. The following findings suggest that genetic alterations in specific tumour genes are associated with ICB responsiveness. By summarizing these genes and signalling pathways, we attempt to reveal the regulatory network of tumour cells for ICB response (Fig. 2).

Lysine methyltransferase (KMT2D) encodes a histone H3K4 methyltransferase and Kmt2d mutation will increase activation of transposable elements, immune infiltration and IFNγ-stimulated antigen presentation.^104^ KMT2D harbours frequent somatic point mutations in tumours and has been identified as a tumour suppressor in multiple solid cancers.^105–108^ Mechanistically, KMT2D loss causes pharmacological inhibition of glycolysis and promotes tumourigenesis through the insulin growth factor (IGF) pathway.^106,109^ KMT2D epigenetically activate PI3K/AKT pathway in prostate cancer and breast cancer.^107,108^ Classical PI3K-Akt- mammalian target of Rapamycin (mTOR) pathway provides important support for antitumour metabolism and ICB response. Frequent gene mutations, such as PIK3CA, PIK3R1, PTEN, AKT, TSC1/2, LKB1, and MTOR, in this pathway are the cause of tumourigenesis and one of the main targets for treatment.^110^ In the area of drug resistance, upregulation of RTKs (HER3, EGFR, IGFR1) and loss of PTEN expression may be the initiator of the activation of the PI3K pathway.^111^ FAT1 alteration and PTEN depletion are the main genetic alteration of rectal neuroendocrine tumours influencing the response to ICB.^112^

DNA damage repair (DDR) defectiveness due to tumour gene mutations will lead to the accumulation of both single-stranded DNA (SSB) and double-stranded DNA (DSB). Inhibition of the DDR pathway can improve the response to ICB in solid tumours.^113,114^ Ataxia telangiectasia mutated (ATM) protein participates in sensing DSBs. Inhibition of ATM will promote mitochondrial DNA (mtDNA) leakage and activate the cGAS/STING pathway in murine cancer cells.^115^ ATM blockade can also increase SRC-dependent type I IFN signalling activation and ICB sensitivity in pancreatic adenocarcinoma.^116,117^ In another hand, PARP inhibitors are synthetically lethal with BRCA mutations.^118^ A truncating mutation in BRCA2 other than BRCA1 is associated with a superior response to ICB.^119^ Another pan-cancer analysis also confirms the association between BRCA2 mutation with pembrolizumab sensitivity.^120^

It is unilateral to judge ICB response only by TMB and PD-1 expression in lung cancer, which is closely related to oncogene alterations. BRAF-mutant NSCLS patients have higher TMB, higher PD-L1 expressions, and more benefits from ICB. On the contrary, EGFR-mutant, HER2-mutant, ALK, ROS1, RET, and MET alterations in NSCLC have limited benefit from ICB.^121^ EGFR-mutant NSCLC performs a non-inflamed immune phenotype due to PKCδ, a gatekeeper of immune haemostasis. Ablation or blockade of PKCδ will promote ICB sensitivity significantly.^122^

Lysine demethylase 5B (KDM5B) suppresses endogenous retroelements and promotes immune evasion via recruiting SET domain bifurcated histone lysine methyltransferase 1 (SETDB1). Depletion of KDM5B may induce robust adaptive immune responses and overcome resistance in mouse melanoma models.^123^ These preclinical studies provide different directions for uncovering resistance but still need to be further confirmed in large sample populations.

### Tumour gene mutations associated with ICB resistance

Alternate promoter burden (APB) high tumours are confirmed with significantly poorer PFS and resistance in gastric cancer patients because of lower T-cell proportion and almost no infiltration into the TME.^124^ Mutations of key genes involved in the antigen-presenting (MHC I, β2M) and IFN-γ signalling pathway (JAK1/2, IFNγR1/2) may lead to loss of tumour antigen and insensitivity to interferons in tumour cells. Deep mutagenesis of JAK1 and missense mutations altering the INF-γ pathway have been highlighted in colorectal cancer patients refractory to ICB.^125^ Loss of Polybromo-1 (PBRM1) in renal cell carcinoma (RCC) reduces the interaction between Brahma-related gene 1 (BRG1) with IFNγ2, leading to decreased STAT1 phosphorylation and IFN-γ target genes.^126^ Knockdown of IFN-γ receptor 1 (IFNGR1) will lead to primary resistance to anti-CTLA-4 therapy in the mouse model. Copy-number alterations (CNAs) of IFNGR1/IFGR2, IRF1, and interferon-receptor-associated Janus kinase 2(JAK2), as well as amplification of SOCS1 and PIAS4, have been identified in non-responders with metastatic melanoma patients through whole-exome sequencing (WES).^127,128^ Mutations of JAK1/2 and beta-2-microglobulin (B2M) have also been identified in PD-1 blockade resistant melanoma patients through WES.^129,130^ Abundant copy-number alterations, B2M loss, and phospholipase A2 group IID (PLA2G2D) overexpressed correspond with adaptive ICB resistance in another pan-cancer analysis.^120^

STK11/LKB1 mutations have been identified as the unique marker significantly associated with PD-L1 negative lung adenocarcinoma patients. KRAS-mutant lung cancers are resistant to PD-1/PD-L1 blockade due to the suppression of STING associated with LKB1 loss.^131,132^ BRCA1-deficient ovarian cancers mediate immune resistance through transcriptional reprogramming and cell-intrinsic inflammation via the STING pathway.^133^

### Tumour gene mutations associated with ICB-related adverse events

The significant association between irAEs and tumour mutational burden has been verified in a large cohort of solid cancer patients no matter during CTLA-4 or PD-1/PD-L1 blockade therapier.^134–136^ Overall, the majority of irAEs are not tumour-specific or organ-specific because of the immune imbalance between excessive T cell activation and over-suppression of Treg cells, resulting in persist release of proinflammatory cytokines.^137^ From the perspective of tumour gene mutations, there exists a high similarity between tumour neoantigens and autologous healthy tissue, so ICB can produce cross-reactivity beyond anti-tumour immunity on healthy cells and produce tumour-specific or organ-specific irAEs as a result. Complex interplay with unclear mechanisms between genetic alterations and TME are thought to involve in this kind of breakdown of immune tolerance.^138^

## Complex mechanistic networks of drug resistance to ICB therapy

In an excellent review, key biological processes related to resistance are summarized into oncogenes, oncoproteins, loss of antigens, dysfunctional T-cell, lack of infiltrating TME, deregulated tumour immunometabolism, and genetic and epigenetic dysfunction.^139^ Established immune-based mechanisms include loss of neoantigens or PD-L1, defects in antigen presentation signalling, dysfunction of the local immune, and T cell exclusion. Mechanisms that lack sufficient clinical evidence but are at the forefront of research include alterations in metabolism, epigenetic regulation, and gut microbiota activation.^140^ Response to anti-PD-1 and clinical benefit has been found in nine PD-1-refractory melanoma patients when combining faecal microbiota transplant before anti-PD-1 therapy.^141,142^ As the most effective ICB drugs at present, we take anti-PD-1/ anti-PD-L1 drug resistance as the starting point to discuss the specific mechanism of immune resistance. In this paragraph, we focus on updated mechanisms from the perspective of DCs, T cells, tumour immunosuppressive microenvironment (TIME), and metabolic alteration respectively.

### ICB resistance and antigen presenting cell dysfunction

DCs are essential antigen-presenting cells (APCs) that present both endogenous and exogenous antigens to T cells and maintain immunity and tolerance balance.^143^ After different stimuli, DCs differentiate and mature, and then exercise different immune regulatory functions. According to different functions, DCs are divided into the conventional DC (cDC) subsets, the plasmacytoid DC subsets, inflammatory DC subsets, and Langerhans cells. In this section, we focus on cDCs and mature DCs enriched in immunoregulatory (mDCregs). Moreover, cDCs are divided into cDC1 (antigen cross-presentation to CD8^+^ T cells) and cDC2 (antigen presentation to helper CD4^+^ T cells). Cross-talk among DCs, immune cells, and tumour cells within the TME is extremely complicated. Briefly, exposed tumour antigens recruit and promote cDCs differentiation, followed by DCs migration to lymph node and T cells activation.^144,145^ On the opposite, mDCreg produces IL-4 and inhibits the functions of DCs and T-helper-1(Th1) cells.

**Fig. 3:**
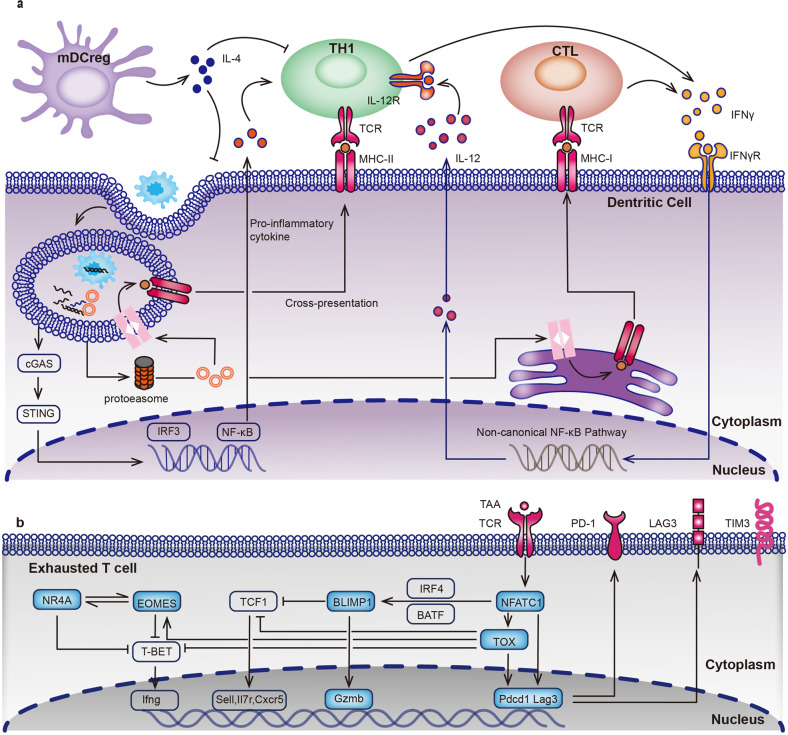
Transcriptional regulation of dendritic cells (DCs) and exhausted T cells (T_EX_). **a** Cell phagocytosis is triggered and results in phagosomal degradation in the cytoplasm of DC cells. Cyclic GMP-AMP synthase (cGAS)-stimulator of interferon genes (STING) signalling is activated and releasing type I interferons (IFNs), mainly comprised of IFN-α and IFN-β. Antigens processed are loaded onto MHCs, which subsequently promote the activation of Th1 cells and CTL cells. IFN-γ released by these activated T cells will stimulate the non-canonical NF-κB pathway and release IL-12. Mature immunoregulatory DCs (mDCreg) produces IL-4 and inhibits the functions of cDCs and Th1 cells. **b** Transcriptional regulation of T_EX_ cells. T cell receptor (TCR) responsive network of transcription factors, including the nuclear factor of activated T cells, cytoplasmic component 1 (NFATC1), thymocyte selection-associated high mobility group box protein (TOX), and B lymphocyte-induced maturation protein 1 (BLIMP1) are overexpressed in T_EX_ cells. NFATC1 and TOX promote the expression of PD-1 (encoded by *Pdcd1*) and LAG-3 (encoded by *Lag3*). Highly expressed BLIMP1 induces granzyme B (Gzmb) expression and represses TCF1 expression. Furthermore, TOX induces eomesodermin homologue (EOMES) and nuclear receptor subfamily 4 group A (NR4A) expressions. All of them can inhibit T-bet, which results in the decrease of IFN-γ releasing. IRF4 interferon regulatory factor 4; BATF basic leucine zipper transcriptional factor ATF-like

The cGAS-STING pathway induces type I interferons (IFNs) releasing and activating innate T cell response. Studies have shown the ability to overcome anti-PD-1 resistance through a stimulator of interferon genes (STING) agonist.^146,147^ Type I IFNs, mainly comprised of IFN-а and IFNβ, are involved in multiple processes of antitumour immunity. For instance, type I IFNs support CD8^+^ CTL survival, increase the cytotoxicity of both CD8^+^ T cell and NK cell, increase inflammation, and decrease Treg cell functions.^148^ On the other hand, cDC1s can be activated and released IL-12 after receiving IFN-γ signal stimulation from anti-PD-1 mAbs blocked T cells. Non-canonical NF-κB pathway will be activated together with overexpression of CD40, BIRC2, MAP3K14, NFKB2, and RELB genes.^149^ In a PD-L1 blockade resistant mouse model, IL-12 mRNA therapy can activate Th1 transformation to TME and promote antitumour immunity which supports the bridging role of dendritic cells in antitumour immunity.^150^ In addition, cDC1 accumulation into the TME depends on NK cells producing CC-chemokine ligand 5 (CCL-5) and XCL1.^151^ Additional intratumoral delivery of CCL-5 can overcome IL-12 therapy or PD-1 blockade failure.^152^ IL-12 is also involved in NK cell stimulation and exhausted CD8^+^ T cell reinvigoration against anti-PD-1 resistant.^153^ Of course, IL-12 is only a representative cytokine of the family, other cytokines, IL-6, IL-15, IL-18, IL-23, and IL-27 also play irreplaceable roles in anti-tumour immunity or ICB resistance.^154^ For instance, high IL-6 level is associated with PD-1 resistance in gastric cancer patients.^155^ IL-15 together with IL-12 are essential for generating CXCR6^+^CD49a^+^ NK cells whose low infiltration into the TME presents PD-1 blockade resistance.^156^ IL-18 is also involved in inducing PD-1 expression in human NK cells.^157^ IL-23 is a kind of Th17-promoting cytokine and decreases the expression of IL-12 through an IL-6/CAAT/enhancer-binding protein β/IL1β-dependent manner.^158^ IL-27 can induce the expression of Th17-promoting cytokines such as IL-6 in mice.^159^ The complex regulatory network of cytokines is crucial in the resistance to immune checkpoint inhibitors (Fig. 3a).

### ICB resistance and T cell exhaustion

Metabolic dysfunction of highly-functional T cells also influences anti-tumour activity and resistance.^160^ When the tumour antigens persist, they will trigger a series of inhibitory transcription mechanisms by the T cell receptor (TCR) responsive network and lead to T cell exhaustion. T cells may differentiate into self-renewing precursor exhausted T (T_PEX_) and terminally exhausted effector T (T_EX_).^161^ Transcription factors NFAT component 1 (NFATC1), basic leucine zipper transcriptional factor ATF-like (BATF), interferon regulatory factor 4 (IRF4), and thymocyte selection associated high mobility group box protein (TOX), promote PD-1 expression in both T_PEX_ and T_EX_ cells.^162–165^ The main difference between T_PEX_ and T_EX_ is the expression of T cell factor 1(TCF-1) and B lymphocyte-induced maturation protein 1 (BLIMP1) which repressed each other.^166–168^ In T_EX_ cells, highly expressed BLIMP1 induces granzyme B (Gzmb) expression and represses TCF1 expression.^169^ Meanwhile, sustained expression of TOX induces eomesodermin homologue (EOMES) and nuclear receptor subfamily 4 group A (NR4A) expressions.^170^ All of them can inhibit T-bet, which results in the decrease of IFN-γ releasing. The epigenetic regulation of T_EX_ still needs further exploration.^171^ T_EX_ cells will no longer respond to the anti-PD-1 drugs and eventually move towards overstimulation-induced cell death (Fig. 3b).^172^

It is difficult to distinguish T cell anergy from terminal T cell exhaustion, which makes T cell “dysfunctional” more representative of the phenomenon of non-response to PD-1/PD-L1 blockade.^173^ Typical mechanisms to explain this phenomenon include terminal differentiation of T cell,^174^ activation-mediated cell death,^175^ senescence,^176^ burned-out,^177^ and impaired metabolism.^173,178^ T cell exhaustion and uncontrolled tumour are mutually causal, and how to overcome immune checkpoint inhibitor resistance can be found from the exploration of the mechanism of reversal T cell exhaustion during the period in T_PEX_.

### ICB resistance and tumour immunosuppressive microenvironment

The TME can be classified into four types based on TMB and T cell inflamed gene signature. Suppressive immune cells (MDSCs, Tregs, TAMs), immunosuppressive cytokines (VEGF, TGF-β, PGE_2_), defective vasculature and stromal cells constitute the TIME together and induce suppressive metabolities.^179^

Firstly, the rapid tumour cell proliferation through glycolysis creates lactate accumulation and an initial hypoxic environment at the tumour site. The hypoxia-inducible factor-1 (HIF-1α) pathway may be activated and modulates aerobic glycolysis and the Warburg effect. The Warburg effect refers to the specific glycolysis metabolism which causes cell proliferation in tumours.^180^ Metabolisms such as hypoxic TME (O_2_ pressure <10 mmHg) may induce immunosuppression, immunoresistance, and multiple biological processes.^181^ Bclaf1 is upregulated under hypoxia and binds to HIF-1α as a transcriptional target. Therefore, regulating Bclaf1 can directly enhance HIF-1α stability and promote tumour progression.^182^ These combined metabolic alterations generate the energy of CTL, recruitment of Tregs, and MDSCs, the polarization of immune-suppressive M2 macrophages, and immunotherapy resistance.^183^ The adenosine signalling and kynurenine metabolites are employed by both MDSCs and tumour cells. Adenosine produced by the ectoenzymes CD39 and CD73 may inhibit the cytotoxicity of effector T cells and enhance the functions of Treg cells. On the other hand, MDSCs and TAMs produce arginase 1 and IDO, which metabolise tryptophan into kynurenine and promote the functions of immunosuppressive cells.^2^

Immunosuppressive cytokines VEGF produced by Treg cells will inhibit the activation and development of CTL directly or inhibit APC mature and CTL priming indirectly.^184,185^ Tumour neovascularization further aggravates the hypoxic and low PH environment, which in turn reduces the infiltration of effective T cells.^186^ Other stromal cells, for instance, cancer-associated fibroblasts (CAFs) contribute to anti-PD-1 or anti-PD-L1 resistance.^187^

## Clinical dilemmas of ICB

### Broad spectrum indications and resistance for ICB-based immunotherapy

The indications of ICB are added or removed as new drugs come along, and at present, it has covered dozens of cancer types. We have summarized the initial and recent indications of all the nine FDA-approved ICB drugs according to their updated medication guide (www.accessdata.fda.gov) (Table 1). Recent common indications include melanoma, non-small cell lung cancer (NSCLC), renal cell carcinoma (RCC), malignant pleural mesothelioma (MPM), oesophagal squamous cell carcinoma (ESCC) and colorectal cancer (CRC). Single-agent treatment has already been recommended as first-line treatment in NSCLC patients (Atezolizumab or Pembrolizumab), metastatic or with unresectable, recurrent HNSCC (Pembrolizumab), and recurrent or metastatic cervical cancer (Pembrolizumab). Ipilimumab (YERVOY) is recommended as the first-line treatment combined with nivolumab (OPDIVO) among patients with advanced melanoma, RCC, unresectable MPM, metastatic NSCLC expressing PD-L1 (≥1%) and unresectable advanced or metastatic ESCC. After nearly a decade of clinical research, the indications of ICB drugs have gradually become more accurate. We need to notice that in the recent indications of KEYTRUDA, microsatellite instability-High (MSI-H), mismatch repair deficient (dMMR), or tumour mutational burden-high (TMB-H) solid tumours are included but with limitations of use. The qualifying conditions of the indications seem to be transformed from tissue origin to molecular characterization of tumour cells gradually.

**Table 1:** FDA-approved immune checkpoint inhibitor drugs and their indications

Product	Target	Year	Initial indications	Recent indications	Most common adverse reactions^a^	BLA^b^
YERVOY (Ipilimumab)	CTLA-4	2011	Advanced melanoma (unresectable stage III and IV) in patients who have received prior therapy	RCC, CRC, HCC, NSCLC, MPM, EC	• Colitis, diarrhoea, fatigue, pruritus, rash (reported in ≥5% of patients) • Decreased appetite, insomnia, headache, nausea, pyrexia, vomiting, and weight loss (reported in ≥5% of patients at the 10 mg/kg dose)	125377
KEYTRUDA (Pembrolizumab)	PD-1	2014	Unresectable or metastatic melanoma and disease progression following ipilimumab or a BRAF inhibitor (if BRAF V600 mutation positive)	NSCLC, HNSCC, cHL, PMBCL, UC, MSI-H/dMMR, CRC, GC, EC, CC, MCC, RCC, EMC, TMB-H, cSCC, TNBC	• Fatigue/asthenia • Pain (abdominal/back/headache/musculoskeletal pain/arthralgia) • Rash, pruritus • Constipation, diarrhoea, decreased appetite, nausea, vomiting	125514
OPDIVO (Nivolumab)	PD-1	2014	Unresectable or metastatic melanoma and disease progression following ipilimumab or a BRAF inhibitor (if BRAF V600 mutation positive)	NSCLC, MPM, RCC, cHL, HNSCC, UC, CRC, HCC, EC, GC, GJC, EAC	• Pyrexia or infection (upper respiratory tract/urinary tract) • Cough, dyspnea • Hypothyroidism	125554
TECENTRIQ (Atezolizumab)	PD-L1	2016	Locally advanced or metastatic urothelial carcinoma who have disease progression within 12 months of neoadjuvant or adjuvant treatment with platinum-containing chemotherapy	NSCLC, ES-SCLC, HCC, melanoma		761034
BAVENCIO (Avelumab)	PD-L1	2017	Metastatic Merkel cell carcinoma (age older than 12 years)	UC, RCC	• Common: decreased appetite, diarrhoea, fatigue, infusion-related reaction, musculoskeletal pain, nausea, rash • MCC: peripheral oedema • UC: urinary tract infection. • RCC (with axitinib): abdominal pain, cough, dysphonia, dyspnea, headache, hepatotoxicity, hypothyroidism, hypertension, mucositis, palmar-plantar erythrodysesthesia	761049
IMFINZI (Durvalumab)	PD-L1	2017	Locally advanced or metastatic urothelial carcinoma who have disease progression within 12 months of neoadjuvant or adjuvant treatment with platinum-containing chemotherapy	NSCLC, ES-SCLC, BTC	• NSCLC: cough, dyspnea, fatigue, pneumonitis, rash, upper respiratory tract infections • SCLC: alopecia, fatigue/asthenia, nausea	761069
LIBTAYO (Cemiplimab-rwlc)	PD-1	2018	Metastatic cutaneous squamous cell carcinoma or locally advanced CSCC who are not candidates for curative surgery or radiation	BCC, NSCLC	• Diarrhoea, fatigue, musculoskeletal pain, rash (reported in ≥15% of patients) • Grade 3–4 laboratory abnormalities: anaemia, hyperkalemia, hyponatremia/hypophosphatemia, increased aspartate aminotransferase, lymphopenia (reported in ≥2% of patients)	761097
JEMPERLI (Dostarlimab-gxly)	PD-1	2021	dMMR recurrent or advanced endometrial cancer	dMMR solid tumours	• Anaemia, diarrhoea, fatigue/asthenia, nausea • Grade 3–4 laboratory abnormalities: decreased lymphocytes/ albumin/ sodium, increased alkaline phosphatase (reported in ≥2% of patients)	761174
OPDUALAG (Nivolumab and Relatlimab-rmbw)	PD-1 & LAG-3	2022	Unresectable or metastatic melanoma (age older than 12 years)	—	• Diarrhoea, fatigue, musculoskeletal pain, pruritus, rash	761234

*irAEs* ICB-related adverse events, *RCC* renal cell carcinoma, *CRC* colorectal cancer, *HCC* hepatocellular carcinoma, *NSCLC* Non-small cell lung cancer, *MPM* malignant pleural mesothelioma, *EC* esophageal cancer, *ES-SCLC* extensive-stage small cell lung cancer, *BTC* biliary tract cancer, *HNSCC* head and neck squamous cell cancer; *cHL* Classical Hodgkin lymphoma, *PMBCL* primary mediastinal large B-cell lymphoma; *UC* urothelial carcinoma, MSI-H/dMMR Microsatellite Instability-High or Mismatch Repair Deficient Cancer, *GC* gastric cancer; *CC* cervical cancer; *MCC* Merkel cell carcinoma; *EMC* endometrial carcinoma, *TMB-H* tumour mutational burden-high cancer; *cSCC* cutaneous squamous cell carcinoma, *TNBC* triple-negative breast cancer, *GJC* gastroesophageal junction cancer, *EAC* esophageal adenocarcinoma, *BCC* basal cell carcinoma

^a^The adverse reactions refer to the most common adverse reactions (reported in ≥20% of patients) when used as a single agent

^b^Biologic License Application (BLA)

As with any conventional treatment, resistance to immunotherapy is bound to exist. Generally, tumour drug resistance can be divided into primary resistance and acquired resistance. The classical drug resistance hypothesis is that spontaneous mutations from the tumour itself will lead to the accumulation of the resistant subclones over time. Alternatively, the dormant primary drug-resistant cancer stems cell subpopulation can lead to tumour regrowth or spread. Unfortunately, drug resistance in the field of immunotherapy is more complex. Factors affecting immune response include dynamic changes in immune activation and depletion, TME, and inflammatory cascade as discussed before. The mechanism of resistance is closely related to the above influencing factors and varies according to different phenotypes. Anticancer immunity phenotypes can be classified as the immune-desert phenotype, immune-excluded phenotype, and the inflamed phenotype according to their immune response.^188^

Clinically, primary resistance is defined as progression after more than 6 weeks (two cycles) but less than 6 months of ICB therapy. There is evidence that anti-PD-1 receptor binding declines beyond 2–3 months after the last dose. Thus, progression within 12 weeks after the last ICB treatment was discontinued in patients who had a benefit from ICB therapy would be considered to be secondary resistance. Relapse in a patient with an initial objective response would be considered to be acquired resistance, regardless of when it occurred.^189,190^ The conventional treatment includes adjustment of the initial administration time of ICB, administration frequency, and combination of multiple immune checkpoint drugs or other novel combination therapies.

### Inseparable immune response and immune-related adverse effects

Immunotherapy is a double-edged sword, sometimes miraculous, but sometimes deadly. FDA labels indicate the adverse effects of different ICB drugs. Common non-atopy reactions (reported in ≥20% of patients) include but are not limited to fatigue, rash, diarrhoea, nausea, pyrexia, pruritus, constipation, cough, and pain (musculoskeletal, abdominal, back pain, headache et al.) (Table 1). Unique adverse effects associated with ICB drugs are termed irAEs due to non-specific immunostimulation.^191^ There is increasing evidence that immune reactivity and side effects are inseparable. The shared T cell receptor sequences may be the main reason for the equivalent immune attack of tumour tissue and normal tissue.^192^ The irAEs vary depending on different immune checkpoint targets. For instance, hypophysitis, colitis, and rash are common side effects with CTLA-4 inhibitors, while arthralgia, hypothyroidism, pneumonitis, and vitiligo are more frequent with PD-1 inhibitors. Thyroid dysfunction is significantly increased when combining PD-1 with CTLA-4 inhibitors. Based on the National Cancer Institute Common Terminology Criteria for Adverse Events (CTCAE), patients with grade 3 or 4 toxicity should stop the application of ICB. Patients with severe toxicity (irAEs Grade≥3) are increased from 10.0% in the PD-1 inhibitor group to 31.0% in the CTLA-4 inhibitor group.^193,194^ Grade≥3 irAEs, including increased lipase and aminotransferase, are increased from 9.7 to 18.9% when combining PD-1 with LAG-3 inhibitors in advanced melanoma patients.^51^ Drug combination or multi-target combination is the main direction of improving the efficacy of ICB immunotherapy, but attention should be paid to controlling adverse reactions in the development of new targets for drug combination with a PD-1 inhibitor.

### Characteristics of immune responses in special populations

Pregnancy is a very special state of the body. Due to the existence and development of foetal allogeneic substances, the immune regulation and cancer attack of the body are in a complex dynamic balance. Th1 cells involved in cellular immunity produce IL-2 and IFN-γ. Meanwhile, Th2 cells involved in humoral immunity produce IL-4, IL-6, and IL-10. Th17 cells involved in the induction of inflammation produce IL-17. During pregnancy, an imbalance between these cytokines shifts from Th1-dominant to Th2-dominant response.^195^ Decreased circulating IL-2 can help maintain the foetal allograft.^196^ As previously mentioned in the non-pregnant population, decreased circulating IL-10 has been discovered earlier with irAEs occurrence in patients receiving anti- CTLA-4 treatment.^197^ Overexpressed circulating IL-17 has been identified with a correlation to grade 3 diarrhoea or colitis when tested at baseline in 35 advanced melanoma patients receiving ipilimumab.^198^ Novel research has integrated 11 circulating cytokines (including GM-CSF, IFN-α2, IL-2, et al.) and defined a toxicity score (CYTOX) system to predict severe toxicity in 147 melanoma patients. The area under the receiver-operating curve (AUC) for the CYTOX score was about 0.7 at baseline or early during anti-PD-1 or anti-CTLA-4 treatment.^199^ There are very limited data on these biomarkers in pregnant people. For pregnant women, teratosis, preterm infants, and pregnancy complications are special immune-related complications. Only two of seven published case reports describe the situation and irAEs of ICB during pregnancy.^200^ Tolerable diarrhoea and liver toxic effects appear but do not force them to discontinue treatment.^201,202^ Therefore, immune-dominated differences in pregnancy can lead to a bias distribution of irAEs, and the detection of the same biomarkers in a large sample population may lead to inconsistent conclusions.

Ageing, sex, and obesity have also been suggested to be significantly correlated with differences in immune responses or irAE occurrence. A clinical predictive model based on data in a cohort of 315 advanced melanoma with pembrolizumab or nivolumab suggests age <65 years and female sex as independent risk factors to the lower immune response to anti-PD-1 treatment.^203^ Consistent with the foregoing, another analysis based on a cohort of 538 patients suggests aged> 60 years respond to more efficient anti-PD-1. Increasing Treg cells have been detected in both mice animal models and clinical tissues. The immune response can be increased when deleting Tregs in animal models.^204^ A meta-analysis based on a cohort of 6096 patients from 11 trials suggests sex-related differences in the immune response to ICB treatment at the same time. ICBs improve both OS and PFS more in males than females (*p* < 0.001) especially in melanoma patients.^205^ These sex-related differences may be related to more excellent adaptive and innate immune responses in females, resulting in specific T cell subpopulations, and an abundance of immunosuppressive infiltration within the tumour microenvironment (TME).^206^ Most of the above conclusions are based on melanoma and lung cancer. However, when the cancer type expands further, the opposite conclusion is suggested. The results of a meta-analysis based on 13,721 patients mainly consisting of NSCLC (9%), melanoma (9%), and clear cell renal cell carcinoma (9%) patients suggest no significant sex-dependent efficacy of ICB using OS as the outcome.^207^ The experience of any grade irAEs has been increased significantly from 25.2% in the proportion of non-overweight patients to 55.6% in the proportion of overweight/obese patients (BMI ≥ 25 kg/m^2^). But fortunately, the immune response of those patients is much stronger.^208^ A retrospective observational study of 374 patients analyses the relationship between obesity with moderate irAEs. Metabolic disease risk is regarded as a necessarily criteria for stratification. Overweight (BMI ≥ 25 kg/m^2^) /low metabolic risk (less than two metabolic diseases) patients own significantly increased risk for irAEs than those with normal weight/low metabolic risk patients. However, there exists no difference of irAEs development between overweight/high metabolic risk group and normal weight/high metabolic risk group.^209^

### Fatal complications and long-term toxicities of ICB

Specific irAEs and pathophysiology mechanisms also differ between ICBs. Both PD-1 and CTLA-4 inhibition will increase T cell activation, abrogate regulatory T (Treg) cell functions, and boost humoral auto-immunity.^210^ Although most of the irAEs are controllable, some fatal side effects cannot be ignored. For instance, although the incidence of cardiac or pulmonary irAEs is less than 1%, it can lead to serious consequences and irreversible clinical outcomes.^211^ Myocarditis is the most common several cardiac toxicity developed within 4 weeks.^212^ Treatment-related pneumonitis may occur till 15.1 weeks after nivolumab treatment.^213^ Haematological irAEs, including autoimmune haemolytic or aplastic anaemia, neutropenia, and immune thrombocytopenia, account for 3–4% of irAEs but may lead to fatal complications.^214^

Long-term implications of ICB-related toxicity are special treatment situations of immunotherapy. Acute toxicities and chromic toxicities often coexist throughout the immunotherapy treatment course.^215^ Chronic irAEs are referred to persist toxicities longer than 12 weeks after receiving ICB drugs and are counted in 43.2% of patients.^216^ Among them, hypothyroidism, type 1 diabetes, and rheumatological toxicities are the most common ones.^217^

### The elusive unconfirmed progressive disease

Different from traditional treatment, immunotherapy has its special mode of antitumour activity. Therefore, how to evaluate the therapeutic effect of immunotherapy and make clinical decisions need to be based on the characteristics of ICB drugs. A guideline named immune Response Evaluation Criteria in Solid Tumours (iRECIST) is now recognized for specific clinical evaluation of immunotherapy during clinical trials. Clearly defining the unconfirmed progressive disease (iUPD) is an important objective of this guideline.^218^ Pseudoprogression (PP) and hyperprogression (HP) diseases are two states of iUPD that are difficult to distinguish in a short-term clinical observation. The assessment period of radiologic is recommended at least 4 weeks and up to 8 weeks after ICB treatment. Patients with PP may achieve long-term efficacy with ICB, while radiological examination can detect an increase in tumour tissue, which can be mistaken for disease progression and lead to discontinuation of treatment. In contrast, HP refers to the real disease progression that occurs without clinical benefit after immunotherapy. The clinical benefit of another atypical response, namely dissociated response, is intermediate, with both effective and ineffective responses occurring in local tumour tissues.^219^

It is essential to delve deeper into the biological nature of these clinical phenomena and develop more accurate assessment strategies. Potential mechanisms of PP include inflammatory infiltration, tumour cell necrosis, and oedema which leads to increased tumour size that is difficult to identify by imaging.^220^ While the heterogeneity of T cell subtypes in the TME and the proteomic domains within the Fc antibody complex could lead to HP occurrence.^221^

Fluorodeoxyglucose (FDG) PET/CT has irreplaceable advantages in the imaging detection of tumour metabolism, which can be used to evaluate the morphological changes of tumours rather than just the size. Tumour response patterns detected through FDG PET/CT include size reduction, cavitation, cystic change, intratumoral haemorrhage, and reduction in vascularity. Certainly, PET/CT provides a rich basis for identifying iUPD, however, no current criteria have been approved in the clinical assessment of PP or HP within 8 weeks of ICB treatment.^222^ Further exploration of this fuzzy zone is an urgent problem to evaluate the effectiveness of ICB treatment.

### Predictive biomarkers for immune response and adverse effect

The development of predictive biomarkers for ICB based immunotherapy has an intimate relationship to drug indications, which attempts to quantitatively describe the heterogeneity of TME from several aspects. Predictive biomarkers are key stratifying factors for population screening and efficacy assessment. It is an indispensable way to transform basic research into clinical application. The current biomarker application mainly involves three aspects: immune biomarkers, genetic biomarkers, and biomarkers in peripheral blood that correlate with clinical outcomes. Among them, PD-L1 expression detection, MSI/dMMR testing, and TMB testing are the three most typical methods^223–225^ (Table 2).

**Table 2:** FDA-approved predictive biomarkers for the immune response of ICB

Biomarker	Detail	Description	Trade name
PD-L1	PD-L1 protein expression	A qualitative immunohistochemical assay used in the assessment of the PD-L1 protein in tumour tissue	Dako PD-L1 IHC 22C3 PharmDx Assay Dako PD-L1 IHC 28-8 pharmDx Assay Ventana PD-L1 (SP142) Assay VENTANA PD-L1 (SP263) Assay
dMMR proteins	MLH1, PMS2, MSH2 and MSH6	A qualitative immunohistochemistry assay used in the assessment of MMR proteins (MLH1, PMS2, MSH2 and MSH6) in tumour tissue by light microscopy	Ventana MMR RxDx Panel
MSI-High	Microsatellite instability-High (MSI-H)	A next-generation sequencing-based detection of genomic signatures including MSI and TMB using DNA isolated from tumour tissue or blood	FoundationOne CDx
TMB	TMB ≥ 10 mutations per megabase	A next-generation sequencing-based detection of genomic signatures including MSI and TMB using DNA isolated from tumour tissue or blood	FoundationOne CDx

*dMMR* deficient mismatch repair, *TMB* tumour mutational burden

PD-L1 expression has been given high expectations to direct assess objective response to their antibodies in early-phage clinical trials because of their high expression in tumour tissues. Regrettably, the data and standardized assay differ among diverse tumours. In the phase 3 trial evaluation of the pembrolizumab treatment effect in PD-L1 positive advanced NSCLC, patients with PD-L1 tumour proportion score (TPS) of 1% or greater were all enroled. Whereas, overall survival of patients was significantly longer in the pembrolizumab group no matter TPS ≥ 50%, 20%, or 1%.^226^ While in another phase III trial comparing the pembrolizumab effect in advanced ESCC, patients were enroled regardless of PD-L1 status. Overall survival of patients was significantly longer in PD-L1 combined positive score≥10% subgroup.^227^ The US FDA has approved individual immunohistochemistry (IHC)-based assays for each drug, respectively Dako 22C3, Dako 28-8, Ventana SP142 and Ventana SP263.^228^ The positive rate of PD-1 expression in NSCLC cells is similar to antibodies 22C3, 28-8 and SP263 detection, but still, needs clinical validation.^229,230^ General commercial assay with optimum cutoff point applicable to multiple tumours is a hot spot for detection development. Moreover, PD-1 expression detection through IHC relies on tumour biopsy specimens. Deep tissue sites or metastatic tissue are not as readily accessible and monitored as superficial tissue. In patients with advanced or distant metastases patients, surgery or biopsy may also increase the risk.

MSI refers to high rates of mutations that result in changes in the length of the microsatellite sequences. dMMR refers to somatic or germ-line mutations in the tumour. Two currently used measurements are PCR for detecting MSI and IHC for detecting dMMR in clinical practice. The recommendation to use five mononucleotide biomarkers, BAT25, BAT26, NR21, NR22, and NR24 in detecting MSI of colon and gastric tumours shows one hundred percent diagnostic sensitivity and specificity.^231^ IHC detection of MMR proteins hMLH1, hMSH2, hMSH6, and/or hPMS2 are also established with diagnostic sensitivity over 90% among multiple solid tumours. However, MSI/dMMR status appears to have no association with anti-PD-1 therapy in acute myeloid leukaemia patients because MMR loss is rare.^232,233^ The MSI/dMMR can predict the responses of ICBs mainly in metastatic colorectal cancer patients using nivolumab plus ipilimumab.^234,235^ Another opinion is that MSI/dMMR is a special subset of TMB because of its unidirectional correlation with TMB. Almost all patients with MSI-H are with high TMB, while the opposite association does not hold. Moreover, MSI/dMMR detection shows no relationship with PD-L1 expression.^236^

TMB is a promising biomarker for multiple ICBs and solid tumours which can be measured both in tumour tissues or in blood. TMB can reflect the frequency of tumour neoantigens and the efficiency of T cell recognition and bring clinical benefits. TMB is also the only FDA-approved biomarker in melanoma. Higher TMB has been shown with relationship to the response to anti-PD-L1 and anti-CTLA-4 in NSCLC, melanoma, and bladder cancers.^237^ However, TMB relies on the replication components and processes of the immune system to achieve this effect.^238^ Mutated processes of leucocyte and T cell proliferation regulation are identified as better interpretable genomic predictors than TMB.^239^ Persistent tumour mutation burden (pTMB) other than TMB is also recommended as a more accurate biomarker of anti-tumour immune responses because it is related to imposing an evolutionary bottleneck under the selective pressure of ICB.^240^ The predictive value of TMB is also controversial because of its ancestry-driven recalibration. TMB-high was significantly associated with improved outcomes only in European ancestries.^241^ Testing of the optimum panel of mutated genes through next generate sequencing (NGS) or whole-exome sequencing (WES) is accurate but expensive. A standardized assay for determining TMB is still lacking. In conclusion, the common dilemma of predictive biomarkers for immunotherapy among multiple tumours is to establish a set of standard testing procedures including combined commercial measurements and quality control standards.

Another important application of big data is the use of multi-omics studies in immunotherapy. The amount of multi-omics data is presented in multiple public databases, for instance, the Cancer Genome Atlas (TCGA), Gene Expression Omnibus (GEO), and UK Biobank et al. LCP1 and ADPGK have been identified as irAE predictors using a bivariate regression model of multi-omics data across multiple cancers.^242^ A novel signature of five GlnLncRNAs has been screened to predict prognosis in glioma.^243,244^ Mutations of seven genes have been detected with better OS in gastric cancer patients receiving anti-PD-1/PD-L1 treatment.^245^ In addition, the deep learning method has also been used to predict the clinical benefits of anti-PD-L1/PD-1 therapy in advanced NSCLC patients.^246^ In conclusion, multi-omics data provide us with multifaceted clues for the development of biomarkers and the design of treatment strategies for immunotherapy.

Due to the vast amount of genetic information generated by single-cell RNA sequencing (scRNA-seq) and mass cytometry technologies, many other predictive markers are emerging. Peripheral blood biomarkers have always been an important piece of information for tumour immunotherapy because they are easy to sample and relatively noninvasive. Detectable biomarkers in peripheral blood include the proportion of particular phenotype immune cells or tumour cells, and circulating tumour DNA (ctDNA). The frequency of CD14^+^CD16^-^HLA^-^DR^hi^ monocyte can be detected with high abundance in the blood of melanoma and predict responsiveness to anti-PD-1 immunotherapy. Increased T cell infiltration and T cell-mediated tumour-killing activity can be promoted in these patients.^247^ Gene signature related to MHC I has also shown a satisfied relationship with anti-PD-1 immunotherapy in melanoma. Inhibitors of CDK4, GSK3B, and PTK2 could enhance tumour response to anti-PD-1 immunotherapy.^248^ Likewise, increased Ki-67^+^PD-1^+^CD8 T cells can be detected in blood samples of NSCLC patients and related to PD-1 targeted therapy responses. The tumour-specific phenotype of these CD8^+^ T cells is CD38^+^Bcl-2 ^lo^ HLA-DR^+^.^249^ Neoantigen-specific T cells can also be detected with a positive correlation of the objective response in peripheral blood of NSCLC patients under atezolizumab treatment.^250^ In the era of urothelial cancer, a proliferation of CD57^+^CD8^+^ T cells has been detected with a relationship to atezolizumab response through scRNA-seq.^251^

Longitudinal ctDNA has been confirmed with a relationship to clinical response or survival in melanoma, colorectal cancer (CRC), and gastric cancer patients with anti-PD-1 treatment.^252–254^ ctDNA carries and transmits tumour burden and genetic mutation information which can help clinicians screen more susceptible populations to ICB drugs. Blood samples of three colorectal cancer patients with dMMR/MSI-H have been used to assess PD-1 and CTLA-4 combined treatment response with ctDNA, CEA, and CA19-9. Results support the utilization of ctDNA as a potential predictive biomarker for immune therapy response.^254^ Another analysis of 18 MSS metastatic CRC patients has identified ctDNA as a predictive biomarker for the therapeutic efficacy of nivolumab immunotherapy.^255^ Meanwhile, undetectable ctDNA, combined with high TMB and a decrease of cell-free DNA can be used in predicting response to checkpoint inhibitors and overall survival in metastatic melanoma patients with anti-PD-1 and anti-CTLA-4 combined treatment.^256^

Non-invasive imaging techniques, for instance, PET imaging and magnetic resonance imaging (MRI) can also help monitor T cell activation and anti-cancer T cell responses. Factors that affect T cell metabolism include accumulation of metabolites, high lactate, and hypoxia in the TME.^257^ 18F-fluorodeoxyglucose (18F-FDG) can monitor increased glucose uptake, at the same time, 18F-fluorothymidine (18F-FLT) can indicate thymidine kinase enzyme activity.^258^ On the other hand, nuclear magnetic resonance (NMR) and magnetic resonance spectroscopy (MRS) can detect energy-related metabolites in tumour tissues which can be used to assess effector T cell density.^259^ These non-invasive assays allow dynamic observation of changes in patient responsiveness. However, clinical samples with big data are still needed for verification.

The occurrence of adverse events can be largely avoided if the risk of irAEs can be predicted before treatment. Consequently, biomarkers for predicting side effects are as important as markers for predicting effectiveness. Risk factors and biomarkers for predicting irAEs have been summarized in a recent review. Although the association of clinical risk factors and underlying diseases with adverse effects is important, specific biomarkers are more urgently needed. Circulating biomarkers such as high neutrophil-lymphocyte ratio, thyroid stimulating hormone (TSH), and troponins are accessible in current practice. Other potential biomarkers in preclinical studies include cytokines, serum proteins, autoantibodies, HLA genotypes, microRNA or gene expression profiling, and microbiota.^260^ The following small sample studies provide some clues for specific cytokines in predicting irAEs. Increased circulating CXCL9 and CXCL10 have been detected in patients with irAEs when tested 2 weeks and 6 weeks post anti-PD-1, PD-L1, or CTLA-4 treatment.^261^ From the above research process, it can be seen that different biomarkers vary with cancer type and checkpoint target. Multiple combinations of predictions may be more accurate and need to be confirmed in larger clinical studies. In general, the development of peripheral blood detection biomarkers is feasible and expected to break through as soon as possible.

## Conventional combination therapies with ICB

Chemotherapy, radiotherapy, targeted therapy and immunotherapy are four important approaches in the field of cancer treatment. The mechanism and clinical research conclusions of the combination of these treatments with ICB have been gradually recognized. In this section, we summarize the new mechanisms and applications of these classical therapeutic applications from the perspective of combination with ICB drugs.

### Chemotherapy or neoadjuvant chemotherapy combined with ICB

Traditional chemotherapy drugs are also called cytotoxic agents to distinguish them from non-cytotoxic drugs such as molecular targeted drugs or immunotherapy drugs. Chemotherapy drugs inhibit tumour cell proliferation and induce tumour cell apoptosis by interfering with the biosynthesis and function of tumour nucleic acid and protein. The mechanisms vary among chemotherapy agents. For example, paclitaxel inhibits tumour cell proliferation by promoting the polymerization of tubulin and inhibiting the depolymerization of microtubules, resulting in mitotic arrest. When platinum drugs enter cells, they form hydrated platinum, which then combines with guanine and adenine in DNA to destroy the DNA structure and interfere with the replication process, thereby inducing cell death. In addition, etoposide can inhibit spindle formation and pemetrexed inhibitors the dihydrofolate reductase and kill tumour cells by interfering with DNA synthesis. From the point of view of the mechanism of the chemotherapy combined with ICB, paclitaxel can also repolarize anti-inflammation population into the pro-inflammatory TAMs as an agonist of TLR4.^262^ Moreover, platinum and taxane chemotherapy, as the most commonly regimen among advanced ovarian cancer, can significantly increase both local T cell oligoclonal expansion and NK cell infiltration during neoadjuvant chemotherapy (NACT).^263^

From the updated specification of the FDA-approved ICBs, we can get the explicit indications and usage of ICB in combination with chemotherapeutic agents. For instance, YERVOY, a human CTLA-4 blocking antibody, is recommended as first-line treatment for metastatic or recurrent NSCLC adult patients with no EGFR or ALK aberrations in combination with nivolumab and platinum-doublet chemotherapy (2 cycles). KEYTRUDA or OPDIVO, a human PD-1 blocking antibody, is recommended as first-line treatment for metastatic non-squamous NSCLC with no EGFR or ALK aberrations in combination with pemetrexed and platinum chemotherapy. KEYTRUDA is also recommended in combination with carboplatin and paclitaxel chemotherapy for metastatic non-squamous NSCLC patients. In the field of HNSCC treatment, KEYTRUDA or OPDIVO is recommended in combination with platinum and FU for metastatic or unresectable, recurrent patients, or as a single agent on or after platinum-containing chemotherapy for these patients with disease progression.^264^ They can also be used in combination with neoadjuvant in triple-negative breast cancer (TNBC), cervical cancer, oesophagal cancer, gastric cancer and gastroesophageal junction cancer. TECENTRIQ or IMFINZI, a human PD-L1 blocking antibody, is used for stage II to IIIA NSCLC (PD-L1 expression ≥1%) as adjuvant treatment following platinum-based chemotherapy, meanwhile, in combination with carboplatin and etoposide for extensive-stage small cell lung cancer (ES-SCLC). Furthermore, IMFINZI can be used in combination with gemcitabine and cisplatin for locally advanced or metastatic biliary tract cancer (BTC). Currently, full-dose chemotherapy is combined with ICB simultaneously. In the future, more advanced approaches in identifying optimal combination strategies, ideal concentration–time profiles and sequence effects will enhance ICB standardization.^265^

### Radiotherapy combined with ICB

Radiotherapy can cause DNA damage-induced tumour cell death, regulate the immunogenicity of tumour cells, expose immunogenic mutations, enhance the expression of neoantigens and increase tumour infiltration of immunostimulatory cells.^266^ The immunomodulatory effects and mechanisms of radiotherapy on chemokines, cytokines, growth factors and immune cells have been well summarized and delineated in a recent review. Radiotherapy is a double-edged sword. On the one hand, it releases proinflammatory factors to transform cold tumours into hot tumours, and on the other hand, it has a certain activation effect on immunosuppressive cells. So far, the best combination strategy is still under exploration.^267^

One major updated purpose of radiotherapy combined with ICB is to enhance the anti-tumour immune response of tumours with low antigen specificity. However, triple therapy seems to be a promising strategy other than two modalities. A synergistic strategy combined with a lysine-specific demethylase 4 C (KDM4C) inhibitor plus radiotherapy and PD-L1 blockade has been identified with efficacy in lung cancer. KDM4C inhibition will induce CXCL10 transcription and enhance the antitumour immune response mediated by CTL.^268^ Dimethylaminomicheliolide (DMAMCL) sensitizes radiotherapy together with PD-L1 blockade and increases tumour infiltrating CD4^+^ and CD8^+^ T cells.^269^ Another schedule combines the immunocytokine L19-IL-2 with radiotherapy and PD-L1 blockade in a poorly immunogenic Lewis lung carcinoma (LLC) model and overcomes resistance through upregulation of inhibitory immune checkpoint molecules on tumour infiltrating T cells.^270^ Current clinical trials are attempting to combine radiotherapy with IL-2 therapy and/or ICB to enhance the antitumour response in cold solid tumours.^269^ Antiangiogenic agents or other agents aiming to turn TIME into an immune-activated TME are also worth expecting.^271,272^

### Targeted therapies combined with ICB

Two main categories of the molecular targeted therapy are monoclonal antibodies and small molecule kinase inhibitors (SMKIs). Molecular targeted therapy achieves anticancer effects mainly through mechanisms such as inhibiting cell proliferation, metastasis and angiogenesis, inducing apoptosis, and reversing drug resistance. However, these agents are available only for patients with targeted driver mutations or aberrations. Furthermore, adverse effects and toxicity due to unexpected cross-reactions with normal tissues and the emergence of intrinsic or acquired resistance affect their effectiveness.^273,274^

In NSCLC treatment, antiangiogenic agents, tyrosine kinase inhibitors (TKIs) for EGFR-mutant or anaplastic lymphoma kinase (ALK) rearrangement patients have significantly improved the clinical prognosis.^275,276^ Antiangiogenic agents, for instance bevacizumab combinded with ICB can improve the theraperutic efficacy at the same time.^277,278^ However, the combination of TKI and ICB is not promising because of higher risk of irAEs and none significant clinical benefits.^279^ It may be a good choice to use ICB after TKI resistance in these patients.

In other tumours, hepatocellular carcinoma patients previously treated with sorafenib are recommended to use dual ICB combination therapy of ipilimumab plus nivolumab or pembrolizumab. Cabozantinib combined with nivolumab is recommended as a first-line treatment in advanced renal cell carcinoma patients. Bevacizumab combined with nivolumab is recommended in the treatment of unresectable or metastatic HCC. Cobimetinib and vemurafenib in combination with atezolizumab are used in unresectable or metastatic melanoma with BRAF V600 mutation positive. Molecular targeted therapy has also been used in RCC and endometrial carcinoma together with pembrolizumab.

### Immunotherapy combined with ICB

Immunotherapy of solid tumours can be broadly divided into ICB, adoptive cell transfer therapy (ACT), tumour-specific vaccines and small molecule immune drugs. Among them, the combination of small molecule immune drugs and ICB has made some progress.

Colony-stimulating factor-1 (CSF-1), also known as macrophage colony-stimulating factor, is one of the most common proinflammatory cytokines leading to various inflammatory diseases. As an immunotherapy target, CSF-1R is the receptor of CSF-1 and participates in the occurrence of solid tumours by regulating the role of TAMs in the tumour microenvironment. In the presence of a CSF-1R inhibitor, TAMs polarization is promoted by granulocyte-macrophage CSF (GMCSF) and IFNγ.^280^ CSF-1R blockade has also been used in combination with insulin-like growth factor-1 receptor (IGF-1R) or PI3K blockade in recurrent gliblastoma multiforme (GBM) patients with significantly prolonged overall survival.^281^ Cabiralizumab, one of the fastest progressing IGF-1R inhibitors, has been used in combination with ICB in advanced solid tumours. Phase 1 clinical trials have finished and confirmed the safety of cabiralizumb in humans.^282,283^ The efficacy of this class of drugs is promising, but mainly for combination therapy rather than monotherapy.

## Research progress on enhancing ICB efficacy

**Fig. 4:**
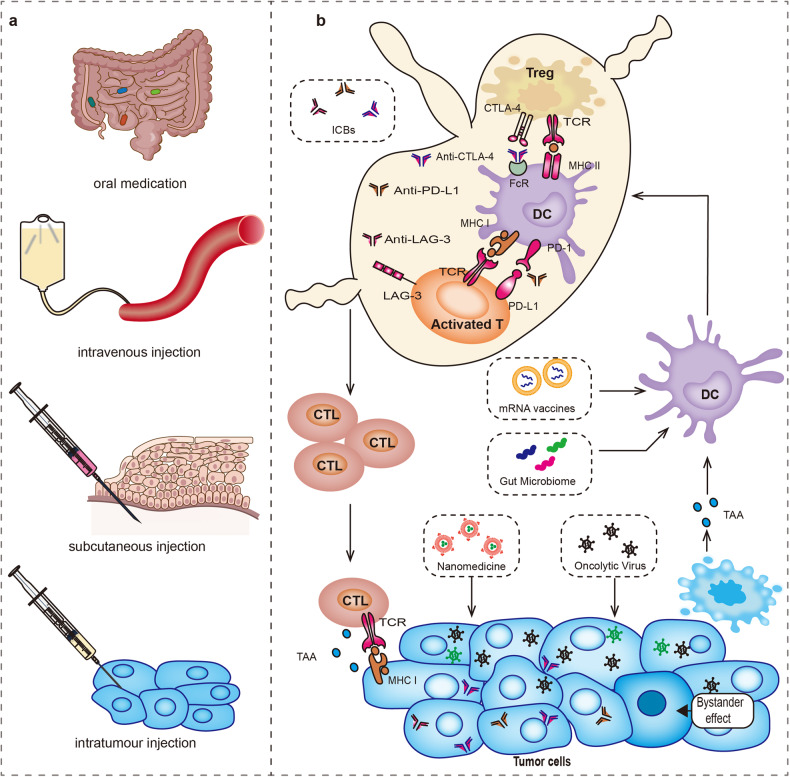
Novel immunotherapy-enhancing combination regimens. **a** The four routes of administration currently used in clinical practice are oral medication, intravenous injection, subcutaneous injection and intratumoral injection respectively. **b** Gut microbiome and mRNA vaccine therapy rely on dendritic cell (DC)-mediated presentation of tumour-associated peptides, antigens, or epitopes derived from tumour lysates to T cells of the adaptive immune system through MHC class II-T cell receptor (TCR) interaction. The cytotoxic T lymphocytes (CTLs) that are subsequently activated interrogate and destroy tumour cells containing tumour-associated antigens presented on MHC class I molecules. Nanomedicine therapy is mainly used to deliver drugs to target organs. Oncolytic virus therapy can directly infect tumour cells to cause lysis and death, or it can translate target proteins in the form of gene editing and play a corresponding tumour-killing role

Due to high cancer heterogeneity, combining ICB with oncolytic viruses (OVs), therapeutic mRNA vaccines, and transplantation of microorganisms have revolutionized immunotherapy treatment and improved efficacy (Fig. 4). Here, we enumerate the feasibility and prospects of each joint application from the perspective of the mechanisms and their respective advantages and disadvantages.

### Complementation of oncolytic viruses with ICB

Basic mechanisms of OV in cancer treatment include infecting tumour cells specifically and lysing them directly, stimulating the innate immune response through pathogen-associated molecular patterns (PAMPs) and damage-associated molecular patterns (DAMPs), releasing tumour-associated antigens (TAAs) or tumour-specific antigens (TSAs) and activating adaptive immune response.^284^ Oncolytic viruses are widely used in drug research and development due to their high antigenicity, effectiveness, and safety of activating immunity. Another advantage of oncolytic viruses is that they can be loaded with diverse functional molecules through gene editing to meet the requirements of different tumour killing. The most representative OV drug is Talimogene laherparepvec (T-VEC), a modified herpes simplex virus-1 (HSV-1) approved in 2015 by FDA.^285^ Although the oncolytic virus is very promising, whether it can be used in combination with ICB to benefit patients is controversial. Some studies have found that the expression of immune checkpoints increases after infection with the virus, which may be related to T cell exhaustion.^286,287^ An increasing proportion of CD8^+^ T cells and helper T cells in non-injected lesions have been detected in phase 2 clinical trials of T-VEC (NCT02366195).^288^ From this perspective, the combination of ICBs and OVs can indeed produce a more durable immune response. Patients treated with intralesional injected T-VEC combined with intravenously injected pembrolizumab have achieved ORR of 62.0% in the phase 1b clinical trial (NCT02263508).^289^ However, the phase 3 clinical trial was terminated because of unsatisfied clinical benefit (NCT02263508). The reason may be that the time interval between OV and pembrolizumab in the clinical trial is not enough to give full play to the immune-activation effect of the oncolytic virus.^290^ Other challenges still exist in many aspects, for instance, the systematic clearance of OV may lead to increased dose and inevitable side effects, the tumour targeting needs to be optimized, and the safety of systemic usage remains unclear. But in any case, oncolytic viruses remain an important strategic development direction for combination applications.

### The recent success of therapeutic vaccines

Therapeutic vaccines targeting TAAs may activate antigen presentation and produce effector T cells. Nevertheless, TAAs are self-antigens that may lead to immune tolerance in advanced tumour patients. Therefore, the combination of ICB with therapeutic vaccines can reduce this immune-suppressive regulation to fully activate the antigen and kill tumour cells. At present, the focus of therapeutic vaccine research is mainly on the activation of various molecular signalling pathways of dendritic cells. The recent success of the mRNA vaccine in coronavirus disease (COVID-19) has paved the way for the development of mRNA vaccines related to cancer therapy. FixVac (BNT111) is a package of four nanoparticulate liposomal RNAs (RNA-LPXs) encoding four TAAs (NY-ESO-1, MAGEA3, tyrosinase and TPTE) specifically prevalent in melanoma. The advantage of FixVac is its ability to active a TLR7-driven type I interferon pathway and profound expansion of antigen-specific T cells.^291^ Meanwhile, except for TAAs, the vaccine has also been designed to augment antigen presentation of immature dendritic cells with an MHC I trafficking domain.^292^ Antigen-specific cytotoxic T-cell responses have been reported alone or combined with a PD-1 inhibitor in the early stage of the phase 1 trial of BNT111 (NCT02410733).^293^ Phase 2 trial of BNT111 combined with cemiplimab is recruiting unresectable melanoma with anti-PD-1-refractory/relapsed patients (NCT04526899). Another tetravalent mRNA vaccine is mRNA-5671 (V941), which targets four KRAS mutations (G12D, G13D, G12C, and G12V). The phase 1 trial of V941 combined with pembrolizumab has been completed in 70 patients with NSCL, pancreatic, and colorectal cancer (NCT03948763). The optimization of the mRNA delivery system and process production are the main bottlenecks in the commercialization of this drug. Multiple phase 1/2 clinical trials are well underway. The effectiveness of therapeutic mRNA vaccine drugs remains to be evaluated over time.

### The miraculous effect of microbiota transplantation

Transplantation of the gut microbiome is currently a hotspot in ICB combined application therapy. Early clinical studies have confirmed the relationship between the gut microbiome and the host’s immune status. Absence or low abundance of the gut microbiome may lead to decreased priming of dendritic cells, anti-tumour T cell activation, and chemotactic factor functions.^294^ Improved anti-tumour responses to PD-1 inhibitor have been observed after microbiome transplantation in epithelial tumour and melanoma patients.^295,296^ 11 bacterial strains have been isolated from a healthy human microbiota and the preclinical model study have confirmed their ability in producing CD8^+^ T cells.^297^ A promising oral microbial drug VE800 consisting of the above 11 bacterial strains is under evaluation combined with nivolumab in an ongoing phase 1/2 study with 54 advanced or metastatic cancer patients (NCT04208958). Transplantation of the gut microbiome is still in the early stage of application in the clinic cohort. The precise mechanisms between gut microbiome with immune response and the complex metabolism regulation still need to be elucidated.^298^

### Application of nanoparticle delivery systems

The application of nanoparticles is emerging as a potential application in drug delivery, phototherapy, and immunotherapy. Multiple cell membranes can be modified, for instance, tumour membrane, immune membrane, erythrocyte membrane, and bacterial membrane.^299^ Engineered cellular nanovesicles (NVs) which present PD-1 receptors on the cell membrane are used earlier to activate anti-tumour immunity through PD-1/PD-1 pathways.^300^ PD-1-MM@PLGA/RAPA is a kind of modified nanomaterial that consist of PD-1 over-expressed macrophage membrane. This kind of strategy helps improve trans-blood-brain barrier delivery and targets PD-L1^+^ tumour cells which upregulate anti-tumour immune response from different aspects.^301^ Another peptide-based self-assembled nanomaterial NLG919@DEAP-^D^PPA-1 aims to modify PD-L1 antagonistic ^D^PPA-1 peptide and improves the efficacy of immunotherapy through targeting PD-L1^+^ tumour cells and inhibiting IDO enzyme.^302^ Engineered leucocyte membrane-coated nanoparticles that encapsulate TGF-β inhibitor and PD-1 antibody may create an immunogenic microenvironment in the tumour tissue and induce lethal ferroptosis through Fe_3_O_4_ magnetic nanocluster composition.^303^ In conclusion, nanoparticles could enhance the efficacy of immune checkpoint inhibitors through engineering and various combination strategies.

## Clinical advances of ICB on solid tumours and related clinical trials

### The updated conclusions of successfully finished clinical trials

Although the efficacy of ICB drugs has been confirmed, there exists considerable variation between different tumours or individuals. We list the results of the signature successful phase 3 clinical trials according to different tumour types. In this way, the effectiveness of the ICB drugs can be examined more intuitively and objectively (Table 3).

**Table 3:** Successfully finished phase 3 clinical trials of immune checkpoint inhibitor drugs among diverse cancer types

Disease	Code	NCT number	Enrolment	Arms	Follow-up	Median PFS/RFS	Median OS	PMID
Untreated unresectable stage III or IV melanoma	—	NCT00324155	681	A: Ipilimumab 10 mg/kg (Q3W, −22 weeks) + Dacarbazine 850 mg/m^2 (Q3W, −22 weeks) B: Dacarbazine 850 mg/m^2 (Q3W, −22 weeks)	5-year	–	A: 11.2 months B: 9.1 months	25713437
Complete resection of high-risk stage III melanoma	EORTC-18071	NCT00636168	1211	A: Ipilimumab 10 mg/kg (Q3W, −3 years) B: Placebo	5-year	A: 27.6 months B: 17.1 months	—	27717298
Unresectable or metastatic melanoma	—	NCT01515189	831	A: Ipilimumab 10 mg/kg (Q3W, 4 doses) B: Ipilimumab 3 mg/kg (Q3W, 4 doses)	5-year	—	A: 15.7 months B: 11.5 months	32503946
Untreated, unresectable, or metastatic melanoma	CheckMate-066	NCT01721772	418	A: Nivolumab 3 mg/kg B: Dacarbazine 1000 mg/m^2	5-year	A: 5.1 months B: 2.2 months	A: 34.8 months B: 20.5 months	32997575
Unresectable or metastatic melanoma	KEYNOTE-006	NCT01866319	834	A: Pembrolizumab 10 mg/kg (Q2W, −24 months) B: Pembrolizumab 10 mg/kg (Q3W, −24 months) C: Ipilimumab 3 mg/kg (Q3W, 4 doses)	5-year	A + B: 8.4 months C: 3.4 months	A + B: 32.7 months C: 15.9 months	31345627
Previously treated advanced or metastatic squamous cell NSCLC	CheckMate-017 CheckMate-057	NCT01642004 NCT01673867	854	A: Nivolumab 3 mg/kg or 480 mg B: Docetaxel	5-year	—	A: 11.1 months B: 8.1 months	33449799
Previously treated NSCLC	KEYNOTE-010	NCT01905657	1034	A: Pembrolizumab 2 mg/kg (Q3W, −2 years) B: Pembrolizumab 10 mg/kg (Q3W, −2 years) C: Docetaxel 75 mg/m^2	5-year	—	A + B: 11.8 months C: 8.4 months	34048946
Previously untreated stage IV, programmed cell death ligand 1 (PD-L1) strong expressing NSCLC	KEYNOTE-024	NCT02142738	305	A: Pembrolizumab 200 mg (Q3W, −35 doses) B: Chemotherapy	5-year	A: 7.7 months B: 5.5 months	A: 26.3 months B: 13.4 months	33872070
PD-L1 strong expressing NSCLC	KEYNOTE-042	NCT02220894	1274	A: Pembrolizumab 200 mg (Q3W, −35 doses) B: Chemotherapy	5-year	—	A: 16.4 months B: 12.1 months	36306479
Locally advanced or metastatic NSCLC	OAK	NCT02008227	1225	A: Atezolizumab 1200 mg B: Docetaxel 75 mg/m^2	2.25 years	—	A: 13.8 months B: 9.6 months	27979383
Stage IV non-squamous NSCLC	Impower-130	NCT02367781	723	A: Atezolizumab+Nab-Paclitaxel+Carboplatin B: Nab-Paclitaxel+Carboplatin	Median 18.5 months	A: 7.0 months B: 5.5 months	A: 18.6 months B: 13.9 months	31122901
PD-L1-selected, chemotherapy-naive stage IV NSCLC	Impower-110	NCT02409342	572	A: Atezolizumab B: Chemotherapy	Median 15.7 months	A: 7.2 months B: 5.5 months	A: 20.2 months B: 13.1 months	32997907
Extensive-disease small cell lung cancer	Impower-133	NCT02763579	403	A: Atezolizumab 1200 mg + EP B: Placebo + EP	Median 22.9 months	—	A: 12.3 months B: 10.3 months	31959349
	CASPIAN	NCT03043872	987	A: Durvalumab+Tremelimumab+EP B: Durvalumab+EP C: EP	Median 25.1 months	—	B: 12.9 months C: 10.5 months	33285097
	ASTRUM-005	NCT04063163	585	A: Serplulimab + EP B: Placebo + EP	Median 12.3 months	A: 5.7 months B: 4.3 months	A: 15.4 months B: 10.9 months	36166026
Advanced or metastatic (medically or surgically unresectable) clear-cell renal cell carcinoma	CheckMate-025	NCT01668784	821	A: Nivolumab 3 mg/kg B: Everolimus	5-year	—	A: 25.8 months B: 19.7 months	32673417
Metastatic or locally advanced/unresectable urothelial carcinoma	KEYNOTE-045	NCT02256436	542	A: Pembrolizumab 200 mg (Q3W, −35 doses) B: Chemotherapy	Median 27.7 months	—	A: 10.1 months B: 7.3 months	31050707
Locally advanced or metastatic urothelial bladder carcinoma	IMvigor-211	NCT02302807	931	A: Atezolizumab B: Chemotherapy	Median 33 months	—	A: 8.6 months B: 8.0 months	33902955
Advanced renal cell carcinoma	CLEAR	NCT02811861	1069	A: Lenvatinib 20 mg + Pembrolizumab 200 mg B: Sunitinib 50 mg	Median 26.6 months	A: 23.9 months B: 9.2 months	—	33616314
Unresectable advanced or recurrent esophageal cancer	ATTRACTION-3	NCT02569242	419	A: Nivolumab 240 mg B: Docetaxel/Paclitaxel	Minimum 17.6 months	—	A: 10.9 months B: 8.4 months	31582355
Advanced/metastatic adenocarcinoma and squamous cell carcinoma of the esophagus that have progressed after first-line standard therapy	KEYNOTE-181	NCT02564263	628	A: Pembrolizumab 200 mg (Q3W, −2 years) B: Chemotherapy	16 months	—	A: 9.3 months B: 6.7 months	33026938
Stage IV mismatched repair deficient or microsatellite instability-high colorectal carcinoma	KEYNOTE-177	NCT02563002	307	A: Pembrolizumab 200 mg (Q3W, −35 doses) B: Chemotherapy	44.5 months	A: 16.5 months B: 8.2 months	No significant difference	35427471
Recurrent or metastatic head and neck squamous cell cancer	CheckMate-141	NCT02105636	361	A: Nivolumab 3 mg/kg B: Cetuximab/Methotrexate/Docetaxel	1-year	—	A: 7.5 months B: 5.1 months	27718784
	KEYNOTE-040	NCT02252042	495	A: Pembrolizumab 200 mg (Q3W, −2 years) B: Cetuximab/Methotrexate/Docetaxel	2-year	—	A: 8.4 months B: 6.9 months	30509740
	KEYNOTE-048	NCT02358031	882	A: Pembrolizumab 200 mg (Q3W, −2 years) B: Pembrolizumab 200 mg (Q3W, −2 years) + Chemotherapy C: Cetuximab + Chemotherapy	Median 45 months	—	A: 14.9 months B: 14.7 months C: 10.8 months	36219809
Squamous cell carcinoma Recurrent or metastatic, platinum-refractory cervical cancer	EMPOWER	NCT03257267	608	A: Cemiplimab 350 mg B: Chemotherapy	Median 18.2 months	—	A: 12.0 months B: 8.5 months	35139273
Persistent, recurrent, or metastatic cervical cancer	KEYNOTE-826	NCT03635567	617	A: Pembrolizumab 200 mg (Q3W, −2 years) + Chemotherapy B: Placebo+Chemotherapy	Median 22 months	A: 10.4 months B: 8.2 months	A: 53.0–54.4% B: 41.7–44.6%	34534429
Previously untreated metastatic triple-negative breast cancer	Impassion130	NCT02425891	902	A: Atezolizumab + Nab-Paclitaxel B: Placebo + Nab-Paclitaxel	Median 18.8 months	—	A: 25.4 months B: 17.9 months (PD-L1 positive)	34272041
Previously untreated locally recurrent inoperable or metastatic triple-negative breast cancer	KEYNOTE-355	NCT02819518	882	A: Pembrolizumab 200 mg (Q3W, −2 years) + Chemotherapy B: Placebo + Chemotherapy	Median 44.1 months	—	A: 23.0 months B: 16.1 months	35857659
Triple-negative breast cancer	KEYNOTE-522	NCT03036488	1174	A: Pembrolizumab 200 mg (Q3W, −2 years)+ Chemotherapy B: Placebo + Chemotherapy	Median 39.1 months	A: 84.5% B: 76.8%	—	35139274

*NSCLC* non-small cell lung cancer, *EP* carboplatin or cisplatin plus etoposide, *CCRT* concurrent chemoradiation therapy, *Q2W* once every 2 weeks, *Q3W* once every 3 weeks, *PFS* progression-free survival, *RFS* recurrence-free survival, *OS* overall survival

As the earliest approved drug, the effectiveness of ipilimumab has been confirmed to some extent in melanoma. The reported 5-year survival rate was 18.2%, the median 5-year recurrence-free survival (RFS) was 27.6 months, and the median 5-year OS was 15.7 months when receiving 10 mg/kg ipilimumab in melanoma patients.^304–306^ However, when compared to CTLA-4 inhibitors, PD-1 inhibitors are more effective and have fewer side effects in melanoma patients. Based on the results of KEYNOTE-006 and CheckMate066 trials, the median 5-year OS of melanoma patients has been increased to 32.7 months (pembrolizumab) and 34.8 months (nivolumab).^307,308^

Likewise, PD-1/PD-L1 inhibitors play a dominant role in the field of lung cancer immunotherapy. Multiple clinical trials have confirmed the 5-year safety and efficacy of these drugs in advanced NSCLC patients. The three major drugs with signature completed phase 3 trials are nivolumab (CheckMate017 and CheckMate057), pembrolizumab (KEYNOTE-010, KEYNOTE-024, and KEYNOTE-042), and atezolizumab (OAK, Impower110 and Impower 130). When compared with chemotherapy, the 5-year OS has been increased from 8.1 months to 11.1 months with nivolumab.^309^ Due to the different inclusion criteria and drug doses, the 5-year OS of pembrolizumab ranges from 11.8 months to 26.3 months.^310–312^ Similarly, significant PFS and OS benefits have also been assumed in NSCLC patients with atezolizumab versus chemotherapy.^313–315^

Effective drugs vary slightly among different urinary system tumours, for example, nivolumab can improve OS from 19.7 months to 25.8 months in clear cell RCC patients.^316^ Pembrolizumab can improve OS from 7.3 months to 10.1 months in urothelial carcinoma patients.^317^ Meanwhile, Pembrolizumab combined with Lenvatinib can improve PFS from 9.2 months to 23.9 months when compared to sunitinib in advanced RCC patients.^318,319^ Atezolizumab can improve OS from 8.0 months to 8.6 months in urothelial bladder carcinoma patients.^320^ While, in oesophagal cancer and head and neck squamous cell cancer (HNSCC) patients, nivolumab and pembrolizumab can both improve OS survival.^321–325^ Pembrolizumab can only increase PFS in MSI-H colorectal carcinoma.^326^ Cemiplimab can improve OS from 8.5 months to 12.0 months in cervical cancer patients.^327^ The first-line treatment pattern of recurrent or metastatic cervical cancer with chemotherapy ± bevacizumab has been broken by the KEYNOTE-826 study. The results have shown that the PFS was 10.4 months in the pembrolizumab group and 8.2 months in the placebo group (*p* < 0.001). Overall survival at 24 months was 53.0% in the pembrolizumab group versus 41.7% in the placebo group (*p* < 0.001).^328^ The 2022 NCCN cervical cancer guidelines recommended pembrolizumab + chemotherapy ± bevacizumab as the first-line treatment for recurrent or metastatic cervical cancer.^329^ Atezolizumab or pembrolizumab combined with chemotherapy has been shown with significant efficacy in multiple completed phase 3 clinical trials with triple-negative breast cancer patients.^330–332^

It can be seen from the above studies that the majority of patients have improved OS, while the median PFS has not been improved in some studies. Obviously, a significant improvement in the OS is a much more desirable outcome. However, it doesn’t mean drugs that simply prolong the PFS or immune response are without effectiveness. On the contrary, some phase 3 trials have been terminated because of non-achieved median survival, and perhaps these drugs still have long-term effects and should not be abandoned prematurely.

### In-depth analysis of the causes of failed or terminated clinical trials

In solid tumours, ICB agents have encountered some dilemmas. So far, multiple PD-1/PD-L1 inhibitors have failed in phase 3 clinical trials. Pembrolizumab has failed in second-line liver cancer, second-line TNBC and first-line gastric cancer, meanwhile, nivolumab has also failed in first-line glioblastoma and liver cancer.

The conclusions of phase 3 clinical trials in small-cell lung cancer (SCLC) are quite different. The results of the phase 3 randomized trial of ipilimumab plus etoposide and platinum (EP) show no prolonged OS in 1132 extensive-disease SCLC (ES-SCLC) (NCT01450761).^333^ Checkmate 451, containing 834 ES-SCLC patients, has obtained regrettable results that nivolumab plus ipilimumab cannot prolong OS versus placebo (NCT02538666).^334^ In another clinical trial (Checkmate331, NCT02481830) with 803 relapsed SCLC patients using nivolumab compared to chemotherapy, no significant benefits have been achieved no matter OS or PFS. Pembrolizumab plus EP can significantly improve PFS in ES-SCLC patients, however, the significance threshold of prolonged OS has not been met (NCT03066778).^335^ Fortunately, PD-L1 blockade atezolizumab plus CP/ET has been identified with positive clinical benefits (Impower133, NCT02763579).^336,337^ Meanwhile, durvalumab plus platinum etoposide significantly improves OS in 805 ES-SCLC patients (CASPIAN, NCT03043872).^338,339^ The ASTRUM-005 study, a landmark phase 3 clinical trial of ES-SCLC, has confirmed the efficacy and safety of slolizumab plus chemotherapy (carboplatin-etoposide) versus placebo plus chemotherapy (carboplatin-etoposide). The median OS increases from 10.9 months to 15.4 months, and 24-month overall survival rates increases from 7.9% to 43.1%. Slulizumab is the first anti-PD-1 monoclonal antibody in the first-line treatment of ES-SCLC worldwide.^340^ In conclusion, PD-L1 inhibitors are more beneficial to patients with SCLC than PD-1 inhibitors. The probable reason may be that PD-L1 has a dual channel blocking effect of PD-1/PD-L1 and PD-1/CD80 pathway, which can overcome the problem of low PD-L1 expression in SCLC tumour cells.

### Dual antibody combination and real-world data

The effectiveness of the combination of two ICB drugs is also controversial. Based on better clinical benefits in patients with a combination of dual antibodies compared with the traditional chemotherapy treatment, the combination regimen of ipilimumab and nivolumab has become the first-line recommended drug in NSCLC (NCT02477826), MPM (NCT02899299), and locally advanced or metastatic ESCC (NCT01928394).^341–343^ Neoadjuvant anti-CTLA-4 and anti-PD-1 blockade have been applicated in urothelial cancer with 46% CR and 41% grade 3–4 irAEs among 24 stage III patients.^344^In the clinical trials with advanced melanoma patients, a combination of ipilimumab with nivolumab can improve both the median PFS and OS than nivolumab or ipilimumab alone (NCT01844505).^345^ In contrast, results of another clinical trial continue to recommend the combination of nivolumab plus chemotherapy other than nivolumab plus ipilimumab as the standard treatment in advanced gastro-oesophageal adenocarcinoma patients with PD-L1 combined positive score ≥5.^346^

In the clinical trials of metastatic NSCLC with PD-L1 tumour proportion score ≥50%, neither PFS nor OS can be significantly prolonged for ipilimumab plus pembrolizumab compared with pembrolizumab alone (NCT03302234).^347^ Based on the aggregate clinical data, RFS cannot be improved in a cohort of 1833 advanced melanoma patients when combined with nivolumab plus ipilimumab versus nivolumab monotherapy (NCT03068455).^348^ We can reach a preliminary agreement that compared with the monoclonal antibodies, dual antibody combination is easier to obtain the clinical benefit, but the population and drugs for combination need to be carefully screened.

The application of “big data” in the study of ICB treatment contains aggregate clinical data and real-world data (RWD). Both of them have advantages and disadvantages and they complement each other. Comprehensive clinical annotation and deep insights into molecular mechanisms are equally important and inseparable.^349^ The main difference between RWD and clinical trials is whether they are screened by the population. The real world accommodates more heterogeneous population characteristics. The same rationale applies to the differences between preclinical and clinical studies. The heterogeneity in human populations is much higher than in experimental animal models. As a result, many of the highly anticipated preclinical findings could not be replicated in clinical studies. Therefore, evidence from special animal models (ageing or obese mice) is essential for preclinical studies of the efficacy of immunotherapy.^350^ The first RWD on the effectiveness of ICB in 66 advanced NSCLC patients from north America and eastern European countries have been revealed with comparable effectiveness to clinical trial data.^351^ Another typical RWD study in 501 NSCLC patients from the USA has confirmed longer overall survival benefits in combined immunotherapy than mono-immunotherapy and chemotherapy.^352,353^ PFS data are comparable between RWD and trials from 1950 stage IV NSCLC patients with pembrolizumab or nivolumab treatments in the Netherlands. However, OS data is unsatisfactory for pembrolizumab.^354^ Other real-world studies also reinforce MSI and TMB-high as predictor biomarkers of immunotherapy.^355^

The rationality of clinical trial design has been gradually improved, including the selection of patients, the dosage of drugs, and the indicators of follow-up. The clinical trial of immunotherapy is no longer an era of blind exploratory trials, but an era of discovering more effective drugs and more precise applicable populations through rigorous and reasonable design.

### A summary of promising ongoing clinical trials

There are multiple promising phase 3 clinical trials in progress. Conclusions from early-stage clinical trials of novel immune checkpoint combinations have been described in detail above, here we summarize these promising ongoing phase 3 clinical trials in Table 4.

**Table 4:** Ongoing phase 3 clinical trials of novel immune checkpoint inhibitors or co-stimulators

Trial identifier	Check-points	Estimated enrolment	Disease	Arms	Estimated study completion date
NCT04082364	LAG-3 PD-1	82 (Actual)	HER2^+^ GC/GEJC	A: Margetuximab + Retifanlimab B: Margetuximab + Retifanlimab + Chemotherapy C: Margetuximab + Tebotelimab + Chemotherapy D: Margetuximab + Chemotherapy E: Trastuzumab + Chemotherapy	December 2023
NCT05064059	LAG-3 PD-1	432	PD-1^+^ colorectal cancer	A: Favezelimab + Pembrolizumab B: Regorafenib/ TAS-102	November 2024
NCT05352672	LAG-3 PD-1	1590	Melanoma	A: Fianlimab + Cemiplimab B: Pembrolizumab + Placebo C: Cemiplimab + Placebo	April 2031
NCT03358875	TIGIT	805	NSCLC	A: BGB-A317 B: Docetaxel	July 2023
NCT04256421	TIGIT PD-L1	490 (Actual)	SCLC	A: Tiragolumab + Atezolizumab + CE B: Placebo + Atezolizumab + CE	March 2024
NCT04294810	TIGIT PD-L1	635	NSCLC	A: Tiragolumab + Atezolizumab B: Placebo + Atezolizumab	February 2025
NCT04543617	TIGIT PD-L1	750	ESCC	A: Tiragolumab + Atezolizumab B: Tiragolumab/Placebo + Atezolizumab C: Tiragolumab/Placebo + Atezolizumab/Placebo	December 2025
NCT04736173	TIGIT PD-1	625	NSCLC	A: Platinum-based Chemotherapy B: Zimberelimab C: Zimberelimab + Domvanalimab	June 2026
NCT04746924	TIGIT PD-1	660	NSCLC	A: Tislelizumab + Ociperlimab B: Pembrolizumab + Placebo C: Tislelizumab + Placebo	May 2025
NCT04866017	TIGIT PD-L1	900	NSCLC	A: Ociperlimab + Tislelizumab + CCRT B: Tislelizumab + CCRT C: Durvalumab + CCRT	September 2025
NCT05568095	TIGIT PD-1	970	UGTA	A: Domvanalimab + Zimberelimab + FOLFOX/CAPOX B: Nivolumab + FOLFOX/CAPOX	January 2027
NCT04128696	ICOS PD-1	315	HNSCC	A: Feladilimab+ Pembrolizumab B: Placebo+ Pembrolizumab	April 2023
NCT04266301	TIM-3	530 (Actual)	MDS CMML-2	A: MBG453 + Azacitidine B: Placebo + Azacitidine	January 2027

*GC* gastric cancer, *GEJC* gastroesophageal junction cancer, *ESCC* esophageal squamous cell carcinoma, *NSCLC* non-small cell lung cancer, *SCLC* small cell lung cancer, *CE* Carboplatin and Etoposide, *CCRT* concurrent chemoradiotherapy, *UGTA* upper gastrointestinal tract adenocarcinoma, *HNSCC* head and neck squamous cell carcinoma, *MDS* myelodysplastic syndrome, *CMML-2* chronic myelomonocytic leukaemia-2

## Conclusions

ICBs have played an irreplaceable role in the treatment of advanced cancers. How to give full play to its effectiveness, reduce side effects, accurately evaluate the treatment progress and adjust the combined treatment strategy are the current research hotspots. The complex dynamic balance of immunity and the high heterogeneity of tumours are significant barriers for researchers. The in-depth exploration of the immune resistant mechanism and the accumulation of clinical application experience are two magic weapons for us to further understand the dilemmas and prospects of ICB in cancer treatment. In this review, the classic immune checkpoint targets that have been used in clinics and the emerging targets with great application potential have been listed simultaneously. We have described and highlighted the related signalling pathways/regulatory mechanisms from the perspective of tumour cells, antigen-presenting cells and CD8^+^ T cells. It’s worth noting that the cell category involved in the immune checkpoint therapy response is far greater than that. Furthermore, NK cells, as an important component of the natural immune system, have participating in the regulatory process of dendritic cells, macrophages, T cells and tumour cells. Drugs designed to target NK cells are gaining more acceptance.^356,357^ Even cells of the same category perform different functions in different tissue microenvironments. For instance, the frequency of T_PEX_ and intermediate-exhaust CD8^+^ T cell differs among uninvolved lymph nodes and metastatic lymph nodes.^358^ In-depth basic research on T cell exhaustion and immunosuppressive regulation is still urgently needed.

On the other hand, there are also many difficulties in analyzing the current application of ICBs from the perspective of clinical standardized treatment. Due to the good market prospects, the phenomenon of broadening the indications of ICBs and the insufficient evidence of combination drugs has emerged. The approval and successful implementation of clinical trials related to ICB require more innovative designs and more closely monitored methods for assessing efficacy.^359^ We need to acknowledge that ICBs are not a panacea, and further delineation of benefit populations requires more instructive molecular biomarkers and evidence from large clinical trials.

In addition to the conclusions discussed in this review, the differences in immune regulatory systems between species, the selection of preclinical animal models, the optimization of clinical trial enrolment criteria and observation endpoints, and the standardization of clinical use of approved ICB drugs are all important research directions that need to be taken into account. Innovative high-throughput sequencing technology, and the rapid development of transgenic preclinical animal models and organoids will certainly accelerate and promote the development and clinical transformation of new-generation ICB drugs.^360,361^ Immunotherapy has shifted from an alternative treatment option for patients with advanced cancer to a recommended first-line treatment for early cancer intervention. The clinical patient benefits of such treatment are worth the lifelong efforts of clinicians and scientists.
